# Cortisol regulates neonatal lung development via Smoothened

**DOI:** 10.1186/s12931-025-03104-0

**Published:** 2025-01-18

**Authors:** Shanshan Lu, Yifei Chen, Jiawen Song, Liangliang Ren, Jun Du, Donglai Shen, Jiayin Peng, Yao Yin, Xia Li, Yuqing Wang, Yan Gao, Siman Han, Yichang Jia, Yun Zhao, Yizheng Wang

**Affiliations:** 1 The Brain Science Center, Beijing Institute of Basic Medical Sciences, Beijing, 100850 China; 2 State Key Laboratory of Cell Biology, CAS Center for Excellence in Molecular Cell Science, Shanghai Institute of Biochemistry and Cell Biology, Chinese Academy of Sciences, 320 Yue-Yang Road, Shanghai, 200031 China; 3 Tsinghua-Peking Joint Center for Life Sciences, School of Medicine, Medical Science Building, Tsinghua University, Beijing, 100084 China; 4 National Clinical Research Center for Aging and Medicine, State Key Laboratory of Medical Neurobiology, Huashan Hospital, Fudan University, Shanghai, 200040 China

**Keywords:** Neonatal respiratory distress syndrome, Lung development, Cortisol, SHH pathway

## Abstract

**Background:**

Neonatal respiratory distress syndrome (NRDS), one of the main causes of neonatal death, is clinically characterized by progressive dyspnea and cyanosis 1 to 2 h after birth. Corticosteroids are commonly used to prevent NRDS in clinical. However, the protective mechanism of the corticosteroids remains largely unclear.

**Methods:**

In this study, the simulation of the molecular docking by Autodock, in vitro binding experiments, and Sonic Hedgehog (SHH) pathway examination in cells were performed to study the directly binding of cortisol to Smoothened (SMO). To explore the effect of cortisol action on the SHH pathway on neonatal lung development, we generated a genetic mouse, in which leucine 116 (L112 in human) of SMO was mutated to alanine 116 (L116A, *Smo*^*a/a*^) by the CRISPR-Cas9, based on sequence differences between human and mice. Then, we performed morphological analysis, single-cell RNA sequencing (scRNA-seq) on lung tissue and fluorescence in situ hybridization (FISH).

**Results:**

In this study, we reported that cortisol, the endogenous glucocorticoid, inhibited the sonic hedgehog (Shh)/SMO-mediated proliferation of lung fibroblasts to maintain the normal lung development. Specifically, cortisol competed with cholesterol for binding to the cysteine-rich domain (CRD) in SMO to inhibit the activation of Shh/SMO signaling, a critical signaling known for cell proliferation. Cortisol did not inhibit the activation of SMO when L112 in its CRD was mutated to A112. Moreover, *Smo*^*a/a*^ (L116A) mice exhibited the immature lungs in which over-proliferation of interstitial fibroblasts and reduction in the surfactant protein were evident.

**Conclusion:**

Together, these results suggested that cortisol regulated cholesterol stimulation of SMO by competitively binding to the CRD to regulate neonatal lung maturation in mice.

**Supplementary Information:**

The online version contains supplementary material available at 10.1186/s12931-025-03104-0.

## Background

Neonatal respiratory distress syndrome (NRDS) is a respiratory disease that mainly happens at preterm and late preterm infants. It is one of the main causes of neonatal respiratory failure and death in neonatal intensive care units at present [31]. It is believed that the lack of alveolar surface active substances (surfactant) largely contributed to occurrence of NRDS [39]. Clinically, corticosteroids are commonly used to prevent NRDS, including dexamethasone and betamethasone [27, 60], agents known to promote fetal lung maturation and increase surfactant proteins. However, the preventive mechanism of cortisol to the NRDS clinical appearance remains largely unclear.

The hedgehog (HΗ) family of secreted proteins, including Shh, participate in embryonic cell differentiation and play an important role in the development of tissues and organs, such as the lung [12, 25]. Shh binds to Patched1 (Ptch1) to release Smoothened (SMO), a membrane protein homologous to G-protein coupled receptors, to elicit Gli-dependent transcription [24, 35]. It plays an important role in the morphology of embryonic lungs, the formation of branching structures and the proliferation of mesenchymal cells. It is divided into five stages of lung developmental process in mouse embryo, including embryonic stage, pseudoglandular stage, canalicular stage, saccular stage and alveolar stage [33]. Shh is expressed in respiratory epithelial cells in a complex and variable pattern throughout embryonic lung development from approximately embryonic day 10 (E10) [2, 4, 41, 56]. And its expression is relatively high at the tips of the primary and secondary lung buds, and low at the developing bronchi. Ptch is expressed in the pulmonary interstitia of the embryonic around E11.5 [2, 44, 64]. The overall expression of Shh and Ptch gradually decreases from E15.5 to birth [2]. While SMO is mainly expressed in epithelial and mesenchymal cells from E12.5 to E16.5 [67]. It has been shown that any component anomaly of HH signaling leads to birth defects and pulmonary disease [25, 56]. The transgenic mice with *Shh* overexpression driven by *SP-C* promoter results in an increase of mesenchymal cells proliferation and an absence of typical alveoli, and the transgenic mice die soon after birth [2]. This is similar to the clinical features of NRDS. In our previous research, we found that these synthesized drugs belonged to the group of glucocorticoids (GCs) could inhibit SHH pathway. These included dexamethasone, fluorometholone, prednisolone, methylprednisolone, and betamethasone, all of which have been used in clinical. All of these drugs are synthetic GCs with anti-inflammatory and immunosuppressive properties. Therefore, we asked whether cortisol, as an endogenous glucocorticoid, regulates SHH pathway to control lung development. Here, we found that the L116 mutation of SMO lost the cortisol inhibition of Shh/SMO signaling in mice led to the postnatal death due to lung defect with thickened interstitial parts and reduced alveolar cavities.

## Materials and methods

### Mice

C57BL/6 mice were purchased from SPF (Beijing) Biotechnology Co., Ltd. The *Smo*^*a/a*^ (SMO L116A) mice were generated by CRISPR-Cas9-mediated knock in approach [68]. Briefly, Cas9 mRNA and gRNA (CCTccggaatgccccccgctgct, PAM site capitalized) were in vitro transcribed. Synthesized donor DNA oligo was 90 nucleotides in length, which contained mutant gRNA sequences (c*gc*ccggaatgccccccgctgct, mutations underlined). The mutations disrupted the gRNA PAM site and introduced L116A. The donor DNA oligo, gRNA, and Cas9 mRNA were injected into C57BL/6J embryos, which were transferred into oviduct ampulla of pseudo-pregnant ICR females (JAX, Stock No. 009122). The positive offsprings were backcrossed to C57BL/6J for several generations to establish the line.

The use and care of animals were complied with the guideline of the Biomedical Research Ethics Committee of Institutes for Basic Medical Science, Beijing, China. All animals were raised under reversed 12 h on day and night at 22 ℃ and fed with rodent food and water in a controlled environment. The mice used in this experiment were neonates born after wild-type mating with the genetic mice.

### Histological methods

We collected and numbered the newborn mice and performed local dissection to separate the organs. Then, we fixed these organs in PFA solution according to the mice number. The type of mice was identified by tail genotype, and the tissues were subjected to paraffin sections, following with HE and Masson staining. At least three batches of mice were acquired during the experiment. Histological analysis of the lungs assessed cell numbers and terminal sac space of lung slices, as described previously [37, 69].

### Single-cell RNA-Seq library generation

The neonatal lung was collected at P0, then soaked in the Tissue Storage Solution.

#### Cell capture and cDNA synthesis

Using single cell 3 ‘Library and Gel Bead Kit V3.1(10x Genomics, 1000121) and Chromium Single Cell G Chip Kit (10x Genomics, 1000120), the cell suspension (300–600 living cells per microliter determined by Count Star) was loaded onto the Chromium single cell controller (10x Genomics) to generate single-cell gel beads in the emulsion according to the manufacturer’s protocol. In short, single cells were suspended in PBS containing 0.04% BSA. About 11,411 cells were added to each channel, and the target cell will be recovered was estimated to be about 4,524 cells. Captured cells were lysed and the released RNA were barcoded through reverse transcription in individual GEMs. Reverse transcription was performed on a S1000TM Touch Thermal Cycler (Bio Rad) at 53°C for 45 min, followed by 85°C for 5 min, and hold at 4°C. The cDNA was generated and then amplified, and assessed quality using an Agilent 4200 (performed by CapitalBio Technology, Beijing).

#### Single cell RNA-Seq library preparation

According to the manufacture’s introduction, single-cell RNA-seq libraries were constructed using Single Cell 5′Library and Gel Bead Kit. Single cell capture, library preparation and sequencing were performed by Beijing CapitalBio Technology (Beijing, China). Single cell capture was performed using the Chromium system (10× Genomics) with single Cell 3′ Chemistry V3.1. The libraries were sequenced using an Illumina Novaseq6000 sequencer with a sequencing depth of at least 92,021 reads per cell with pair-end 150 bp (PE150) reading strategy.

### Sc-RNA seq data preprocessing

#### Seurat pipeline

The other clustering method is Seurat 3.0(R package). Cells whose gene number was less than 200, or gene number ranked in the top 1%, or mitochondrial gene ratio was more than 25% were regarded as abnormal and filtered out. Dimensionality reduction was performed using PCA, and visualization was realized by TSNE and UMAP.

#### Enrichment analysis

GO enrichment, KEGG enrichment, Reactome enrichment and Disease enrichment (human only) of cluster markers were performed using KOBAS software with Benjamini-Hochberg multiple testing adjustment, using top 20 markers gene of cluster. The results were visualized using R package.

### Cell culture

NIH-3T3, Mouse Embryo Fibroblasts (MEFs), and HeLa cells were cultured in the medium (Dulbecco’s Modified Eagle Medium, DMEM, Thermo Fisher Scientific) plus 10% fetal bovine serum (FBS, Excell). The MEFs were isolated from E14.5 embryos of pregnant female mice as described previously [7]. Briefly, the head, spine and all the internal organs were removed and discarded, and the remaining tissues were dissociated using eye scissors and incubated in 0.05% trypsin (Sigma) for 30 min with shaking every 5 min. Then, the digestion was stopped by adding a complete medium, filtered, and used for centrifugation. After resuspended, the cells were planted in 25 ml culture flask (CORNING) with MEF medium (DMEM plus 10% FBS) and used for genotyping.

### Whole-cell patch-clamp recordings

HeLa cells cultured on cover glasses at room temperature were used for whole-cell recordings as described previously [18]. Patch pipettes were pulled from borosilicate glass capillaries (1.50 mm OD/0.86 mm ID) by a Flaming/Brown micropipette puller (P-97, Sutter Instruments). The resistance of recording electrodes was 4–6 MΩ when filled with internal solutions containing (in mM): KNO_3_ 140, MgCl_2_ 2.5, EGTA 11, Na_2_ATP 5, and HEPES 10, pH adjusted to 7.3 with KOH. Cells were incubated with extracellular solution (ES) containing (in mM): NaCl 135, KCl 5.4, CaCl_2_ 1.8, MgCl_2_ 1.3, glucose 10, HEPES 10, D-CPPene (NMDA receptor antagonist) 0.03, CNQX (AMPA/KA receptor antagonist) 0.01, bicuculline (GABAA receptor antagonist) 0.02 and UCPH101 (GLAST inhibitor) 0.01, pH adjusted to 7.4 with NaOH. An EPC10_USB amplifier and PatchMaster software were used (HEKA Instruments). The membrane potential was held at -80 mV and inward current was evoked by aspartate (100 µM). The current was recorded under gap-free consecutively. The ES or aspartate dissolved in ES was applied to the cells using the 8-Channel Focal Perfusion System (ALA Scientific Instruments).

### Cloning and transfection

The full length of human SMO was cloned into pCDH-CMV-MCS-EF1-copGFP (CD511B-1) plasmids. The human SMO was then sub-cloned into pcDNA3.1-EF1a-mcs-3flag-CMV-EGFP for the binding experiments. The HeLa cells or HEK293 cells were transiently transfected with these constructs for the respective experiments. Site-directed mutagenesis was made using the KOD plus (KOD-201, TOYOBO). All transfections were conducted using Lipofectamine 2000 (Invitrogen).

### GLI-luciferase assay

NIH3T3 cells harboring the GLI-RE (response elements) were plated at 1 × 10^4^ cells per well into 96-well tissue culture-treated plate (Costar). After 24 h, when the cells reached confluency, the culture medium was replaced with the medium plus sterols, including Betamethasone, Fluorometholone, Methylprednisolone, Methylprednisolone, Prednisolone and cyclopamine, in different concentrations for 30 h. The luciferase activity was measured using the Dual-Luciferase Reporter Assay System (Promega).

### Reverse transcription and real time qPCR

Total RNA extracted using TRI reagent was reverse-transcripted into cDNA using M-MLV reverse transcriptase as the following: 37℃, 30 min; 85℃, 2 min and 4℃, 10 min. The qPCR was performed with Step One Plus Real-Time PCR System (Applied Biosystems, USA) as the following: 95℃, 2 min; 95℃, 10 s and 60℃, 30 s for 40 cycles; 95℃, 15 s; 60℃, 1 min. The results were captured and analyzed with Step One software.

The *β*-actin expression values were used as a control for RNA quantity. Data were shown as the average of a minimum of three biological replicates for each genotype per condition *±* s.e.m.

### Western blot

The cell lysates extracted with 1*×*SDS were electrophoresed on SDS-7.5%PAGE and transferred to PVDF membranes (GE Healthcare Life science). The membranes were blocked with 5% milk in PBS at room temperature for 1 h, then incubated with the primary antibodies against GLI1 (rabbit 1:800, CST) and *β*-actin (mouse 1:1,000, CST) at 4℃ overnight. The second day, after washing the membranes with TBST (GenStar), the membranes were incubated with HRP-conjugated secondary antibodies (Proteintech) at room temperature for 2 h. After fully washing with TBST, the protein bands were visualized by Tanon 5200 (Tanon) using Luminate Crescendo Western HRP Substrate and band density was determined by ImageJ 2.0.

### CCK-8 for cell proliferation

The MEFs were cultured into 96-well tissue culture-treated plates at a density of 3 × 10^5^ cells per well overnight. The cells were then incubated with the medium plus 1% serum for 12 h. The Shh or cortisol were then added into the cultures for the incubation overnight. The next day, after the replacement of the original medium with the prepared medium containing CCK-8, the cells were incubated at 37 °C for 1–3 h. After that, the cells were placed in a microplate analyzer to read the absorbance value at 450 nm.

### SMO purification

The SMO proteins expressed in HEK293T cells transfected with cDNA3.1-EF1a-SMO-3flag-CMV-EGFP were purified by anti-Flag beads as described previously [65]. Briefly, the cells were harvest into 1.5 ml centrifuge tubes and centrifugated after transfection for 48 h. The collected cells were lysed by lysis buffer (in mM: HEPES 20, pH7.4, NaCl 150, plus 1% (w/v) n-dodecyl-β-D-maltopyranoside (DDM, Sangon) and mammalian protease inhibitors) on the ice for 30 min. The lysates were centrifuged at 13,200 rpm at 4℃ for 10 min to collect the supernatant into a new centrifuge tube and incubated with M2 beads (Sigma) at 4℃ for 4 h. Protein-bound beads were washed with the washing buffer (in mM: HEPES 20, pH7.4, NaCl 150, plus 0.2% DDM) and eluted in 100 µl Elution Buffer (in mM: HEPES 20, pH7.4, NaCl 150, plus 0.05% DDM and 0.5 mg/ml 3×Flag peptides) for 30 min. The supernatant was then collected for the sterol binding assay.

### Preparation of sterol biotin beads

The high-capacity neutravidin beads (Thermo) were washed and equilibrated with the washing buffer. The beads slurry was incubated with sterol-biotin probe (Synthesize by WuXi AppTec) at room temperature for 2 h with rotation. The slurry was centrifuged at 1,000×g for 1 min to remove the supernatant. The slurry was washed with the washing buffer, then used for SMO binding assay.

### SMO binding assay

The SMO binding with biotinylated sterol beads and the consequent pull down were conducted as described previously [65]. Briefly, the purified SMO was pre-incubated with the competitor at 4℃ for 1 h before addition of biotinylated sterol beads in the competed inhibition assay. The sterol biotin beads were incubated with the purified SMO and rotated at 4℃ for 2 h for affinity capture. The binding beads were washed with HN buffer (in mM: HEPES 20, pH7.4, NaCl 150, plus 0.05% DDM) and then boiled with same volume 2×SDS sample buffer for 10 min. The SMO levels in the input and binding to the sterol biotin beads were determined by immunoblotting using the anti-Flag antibody (rabbit 1:1,000, Abclonal).

### HPLC

In sterol binding assay, the cortisol was incubated with the biotinylated cholesterol beads. After washing four times, the biotinylated cholesterol beads were boiled in high-salt solution for 10 min to collect the supernatant for the analysis by using HPLC following the vendor protocol (Agilent TC C18, Agilent Technologies). The mobile phases (water: methyl alcohol, 40%: 60%, v/v) were set at a flow rate of 1 ml/min. The sample injection volume was 10 µl and the column temperature was set at 4 °C. Peaks were identified based on the retention time of the standards and confirmed by comparison of the 242 nm wavelength scan spectra.

### RNA FISH and Immunofluorescence

The lungs of mice were fixed in 4% formalin and embedded in paraffin. Cut sections of the lungs were deparaffinized and rehydrated before proceeding to the in-situ hybridization, which was performed according to the manufacturer’s instructions (Advanced Cell Diagnostics, ACD). The mouse probe Mm-Smo (318411, ACD) was used. For PDGFRα and pro-SP-C immunohistochemistry in RNA FISH samples, the sections were blocked with blocking buffer (PBS plus BSA 1% and Triton 0.1%) for 1 h, and then incubated with primary antibodies overnight at 4 °C. After being washed three times, the sections were incubated with anti-rabbit and anti-goat, conjugated to Alexa Fluor 488 secondary antibodies (Molecular Probes) for 1 h at room temperature, and then stained with DAPI (1 mg/ml) for 10 min, followed by the extensive washing. Slides were then dehydrated and mounted using Aqua-Ploy/mount (Polysciences). Confocal images were captured using a 40 × oil immersion objective on the Leica TCS SP8 laser scanning microscope and analyzed using ImageJ/Fiji software. The primary antibodies used were anti-PDGFRα (goat 1:100, R&D) and anti-pro-SP-C (rabbit 1:500, Sigma).

### 5-ethynyl-2’-deoxyuridine (EdU) proliferation assay

Cultured MEF cells were isolated from WT or *Smo*^*a/a*^ mice and 1 × 10^5^ cells per well were seed on round coverslip (SAINING) into 12 well plates overnight. Each well was supplied with the indicated drugs and incubated for 12 h, then treated with 10 µM EdU (Abbkine EdU kit) for another 12 h, and fixed in 4% paraformaldehyde (PFA) for 15 min. Each coverslip was washed three times with BSA Wash Solution and permeabilized in 0.5% Triton X-100 in PBS for 20 min. The coverslips were incubated with Click-iT reaction mixture for 30 min at room temperature in the dark and then washed two times with BSA Wash Solution. Nuclei was labeled with DAPI and finally photographed by Nikon A1 confocal fluorescence microscopy. The percentage of EdU labeled cells was counted by ImageJ.

### Molecular simulation

The pdb file of target protein SMO, 5l7d was obtained from the Protein Data Base (pdb) [3]. The initial preparations of the pdb file, including selecting the needed chains, deleting multiple ligands and non-protein parts, changing molecular graphics and other analyses were performed with the Accelry Discovery Studio.

*(*http://accelrys.com/products/collaborative-science/biovia-discovery-studio/).

The ligand files were retrieved from PubChem compound *(*http://www.ncbi.nlm.nih.gov/pccompound). The PDBQT files were prepared by ADT according to the manual published by Forli et al. [19]. The AutoDock Vina was used for protein-ligand docking [1]. The three-dimensional affinity and electrostatic grid boxes were generated that cover the entire target protein using AutoGrid. The AutoDock Vina parameters were generated by modifying the corresponding numbers of box size and box center according to the manual published by Forli et al. [19]: /vina --config configfile.txt (1). Then, the sites with lowest binding energies were used for further analysis and were visualized by UCSF Chimera package [50]. One corresponding amino acid of each binding result was generated by Accelry Discovery Studio.

Involved algorithm tools were as following:

**AutoDock Vina** AutoDock Vina [1].

**Autodock Tools (ADT)** ADT (Version 4.2) http://autodock.scripps.edu.

**UCSF Chimera package** UCSF Chimera [50].

### Statistical analysis

All experiments were performed with at least 3 biological replicates. Unpaired two-tailed Student’s t test was used for comparison between the two groups. The small sample size and the potential non-normal distribution of the data, we thus used non-parametric tests. Multi-group comparisons were analyzed by one-way and two-way ANOVA Tukey’s multiple comparison test or two-way ANOVA Bonferroni’s multiple comparison test. In all cases, statistical significance, as denoted by (*), was defined as * *p* < 0.05, ** *p* < 0.01, *** *p* < 0.001 and **** *p* < 0.0001, ns., no significance, *p* > 0.05. Data were all expressed as mean ± S.E.M.

## Results

### Cortisol inhibited the SHH pathway

**Fig. 1:**
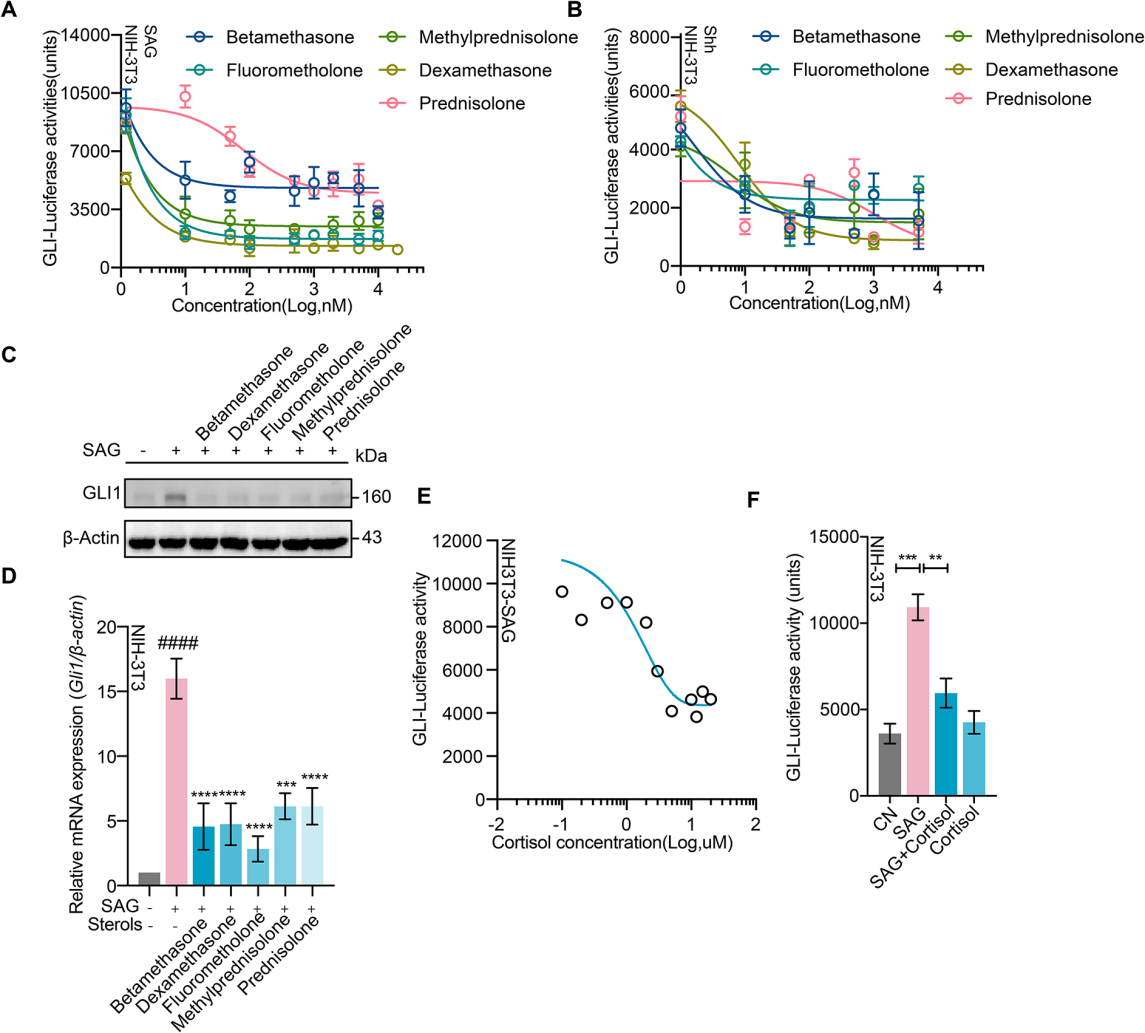
Cortisol inhibited activation of SHH pathway. **A** and **B**, Luciferase activity in NIH-3T3 cells transfected with GLI-luciferase reporter in the presence of SAG (**A**) or Shh (**B**), treated with indicated compounds for 30 h, *n* = 3. **C-F**, Cell quantitative analysis: Representative blotting of total cell lysates of NIH-3T3 cells (**C**) in the presence of the indicated agents using the indicated antibodies. Relative mRNA expression of *Gli1* in NIH-3T3 cells (**D**, *n* = 6) in the presence of the indicated agents. **E**, Luciferase activity in NIH-3T3 cells stimulated with SAG in the presence of different doses of cortisol. **F**, Luciferase activity in NIH-3T3 cells in the presence of the indicated agents, *n* = 3. Unless stated, SAG, Smoothened agonist, 3 µM; Shh, Sonic hedgehog, 500 ng/ml; Cyc, cyclopamine, 10 µM; sterols, 500 nM; cortisol, 1 µM. CN, control. #, compared with CN group; *, compared with SAG group.All western blots performed three or more replicates. Data are represented as mean ± SEM. * *p* < 0.01; ** *p* < 0.05; *** *p* < 0.001; **** *p* < 0.0001, #### *p* < 0.0001. See also Supplementary Fig. 1

We initially studied whether the five synthesized glucocorticoids(GCs)(Supplementary Fig. 1A) indeed affected Shh/SMO signaling. We measured *Gli1*-luciferase activity and *Gli1* expression in NIH-3T3 cells triggered by Smoothened agonist (SAG) in the presence of these five drugs. The SAG-dependent (Fig. 1A) or Shh-dependent (Fig. 1B) *Gli1*-luciferase activity in NIH-3T3 cells was inhibited by these drugs and cyclopamine, a representative antagonist of the SHH pathway [18, 59] (Supplementary Fig. 1B). In addition, the upregulation of both Gli1 protein and mRNA induced by SAG in NIH-3T3 cells (Fig. 1C, D) was suppressed by these drugs. These five drugs alone had no effect on *Gli1* expression (Supplementary Fig. 1C).

Application of cortisol, a physiological GCs with a sterol structure similar to the five GCs (Supplementary Fig. 1A), also inhibited the SHH pathwayas evidenced by downregulating the *Gli1* activity (Fig. 1E, F and Supplementary Fig. 1D) stimulated by Shh or SAG. In the luciferase assays, the IC50 of cortisol inhibition of SHH pathway was close to 1µM (Fig. 1E). We thus used cortisol at 1µM to block SMO signaling in all cellular experiments. Together, these results showed that cortisol inhibited the activation of SHH pathway.

### Cortisol inhibited SMO activation through competing with cholesterol for binding to the CRD

**Fig. 2:**
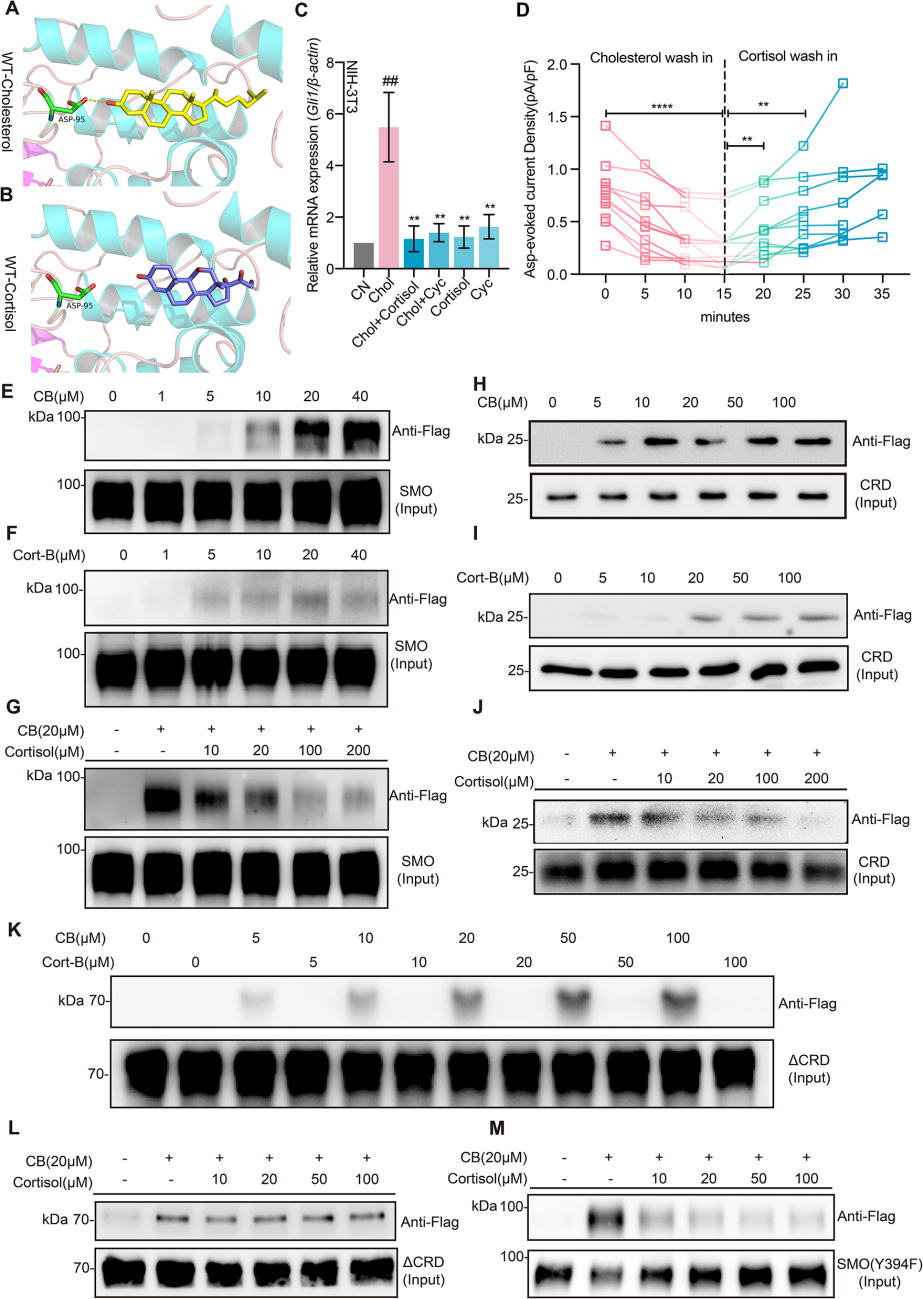
Cortisol competed with cholesterol for binding to the CRD to inhibit SHH pathway. **A-B**, A stable pose for CRD-cholesterol (**A**) and CRD-cortisol (**B**) revealed by molecular simulations. **C**, Relative mRNA expression of *Gli1* in NIH-3T3 cells incubated with cholesterol in the presence or absence of cortisol or Cyc, *n* = 4. **D**, Asp-evoked current density in cultured neurons with cholesterol wash-in for 15 min and then with cortisol wash-in for 20 min, *n* = 11. **E**-**G**, Purified SMO binding to biotinylated cholesterol or biotinylated cortisol. The cholesterol (**E**) and cortisol (**F**) bound to SMO at indicated concentrations. SMO captured on cholesterol beads in the presence of increasing concentrations of free cortisol (**G**). **H**-**J**, Purified CRD domain of SMO binding to biotinylated cholesterol or biotinylated cortisol. The cholesterol (**H**) and cortisol (**I**) bound to CRD at indicated concentrations. CRD captured on cholesterol beads in the presence of increasing concentrations of free cortisol (**J**). **K**, Purified ΔCRD of SMO binding to biotinylated cortisol and cholesterol. **L** and **M**, ΔCRD (**L**) and Y394F SMO (**M**) captured on cholesterol beads in the presence of increasing concentrations of free cortisol. The concentration of sterols provided in the methods was the concentration added to the binding system, not representing the specific binding concentration. Unless stated, Cort, cortisol; WT, wild type; Chol, Cholesterol, 5 µM; Cortisol, 1µM; Mifes, mifepristone, 10 µM; Eple, eplerenone, 10 µM. All western blots performed three or more replicates. All the drugs used in recording and in *Gli1* expression were incubated for 30 min and 36 h, respectively. Data are represented as mean ± SEM. * *p* < 0.01; ** *p* < 0.05; *** *p* < 0.001, ## *p* < 0.05. CB, biotinylated cholesterol; Cort-B, biotinylated cortisol. See also Supplementary Figs. 2 and 3

Studies have shown that cholesterol binds to purified SMO and activates SHH pathway in cultured cells [6, 65]. SMO, a key transducer of the SHH pathway and a drug target, contains an extracellular cysteine-rich domain (CRD), a seven-transmembrane domain (7TMD), and an intracellular part as well [6]. Cholesterol is also important for SMO activation in embryogenesis. Moreover, it binds to the CRD pocket and stimulates SMO activation [5, 6, 22, 23, 28]. Since cortisol has a sterol ring structure similar to cholesterol (Supplementary Fig. 1A), we reasoned whether cortisol inhibits SMO activation through competing with cholesterol to bind to the CRD. A simulation of the molecular docking by Autodock showed that cortisol binding sites on SMO were indeed in the CRD pocket, to where cholesterol also bound (Fig. 2A and Supplementary Fig. 2A). In addition, some cortisol binding residues predicted, such as L108, W109, G111, and L112, were able to bind to cholesterol as well (Supplementary Fig. 2B-D), suggesting that cortisol likely competed with cholesterol for SMO. We then determined the concentrations of cholesterol to activate SHH pathway using the Asp-evoked current protocol [18] and found that the optimal concentration of cholesterol to activate the signaling was 5µM (Supplementary Fig. 2E). Cholesterol was used to stimulate SMO signaling at 5µM in all cellular experiments. Cholesterol enhanced *Gli1* expression in cultured NIH-3T3 cells in a cortisol- or cyclopamine-sensitive manner (Fig. 2C). Similarly, cortisol reversed the reduction in Asp-evoked current induced by cholesterol (Fig. 2D). It did not affect the basal *Gli1* expression (Supplementary Fig. 1D). These results indicated that cortisol acted on activated SMO. Application of mifepristone or eplerenone, which were the inhibitors for glucocorticoid receptors (GRs) or mineralocorticoid receptors (MRs), respectively, did not change the cortisol inhibition on the current density (Supplementary Fig. 2F). These results suggested that cortisol inhibited SMO activation independent of GRs and MRs.

Previous studies have shown that cholesterol can bind to the CRD and 7TMD in the SMO [5, 6, 22, 23, 28]. We then studied whether cortisol could directly bind to the SMO. The cholesterol-biotin with a similar function of cholesterol [65] was designed to minimize the problem of cholesterol solubility and applied in the binding experiments. We made different fragments of SMO (Supplementary Fig. 2G) and performed binding assays using purified SMO or its fragments, biotinylated cholesterol (CB), and biotinylated cortisol (Cort-B) (Supplementary Fig. 2H). As shown in Fig. 2E and F, both CB and Cort-B bound to the SMO in a dose-dependent manner with an observed threshold at 5 µM and as the concentration increased, the cholesterol binding was stronger than cortisol. The CB binding to the SMO was inhibited by cortisol in a dose-dependent manner with a threshold at 10 µM (Fig. 2G). In the assays using the fragments with only CRD domain (CRD) and the fragments with CRD-free domain (ΔCRD), both CB and Cort-B bound to the CRD in a dose-dependent manner (Fig. 2H, I), and the CB binding to the CRD fragments was inhibited by cortisol (Fig. 2J). The CB remained binding to the ΔCRD (Fig. 2K). In contrast, the Cort-B did not bind to the ΔCRD (Fig. 2K). Moreover, cortisol did not affect the CB binding to ΔCRD (Fig. 2L), suggesting that cortisol competed with cholesterol only for binding to the CRD. It has been reported that cholesterol binds to the 7TMD at Y394 site of SMO and Y394 mutation can block cholesterol binding in the 7TMD [28]. However, mutation of the Y394 to F394 did not change CB binding to the SMO (Fig. 2M) and cortisol remained inhibiting the CB binding to the mutated SMO in a dose-dependent manner (Fig. 2M). Moreover, cortisol did not directly bind to cholesterol as shown in the binding assay and high-performance liquid chromatography analysis (Supplementary Fig. 3A, B). All these results suggested that cortisol competed with cholesterol for binding to CRD and inhibited SMO activation.

### L112 of the CRD was essential for cortisol to inhibit SMO activation

**Fig. 3:**
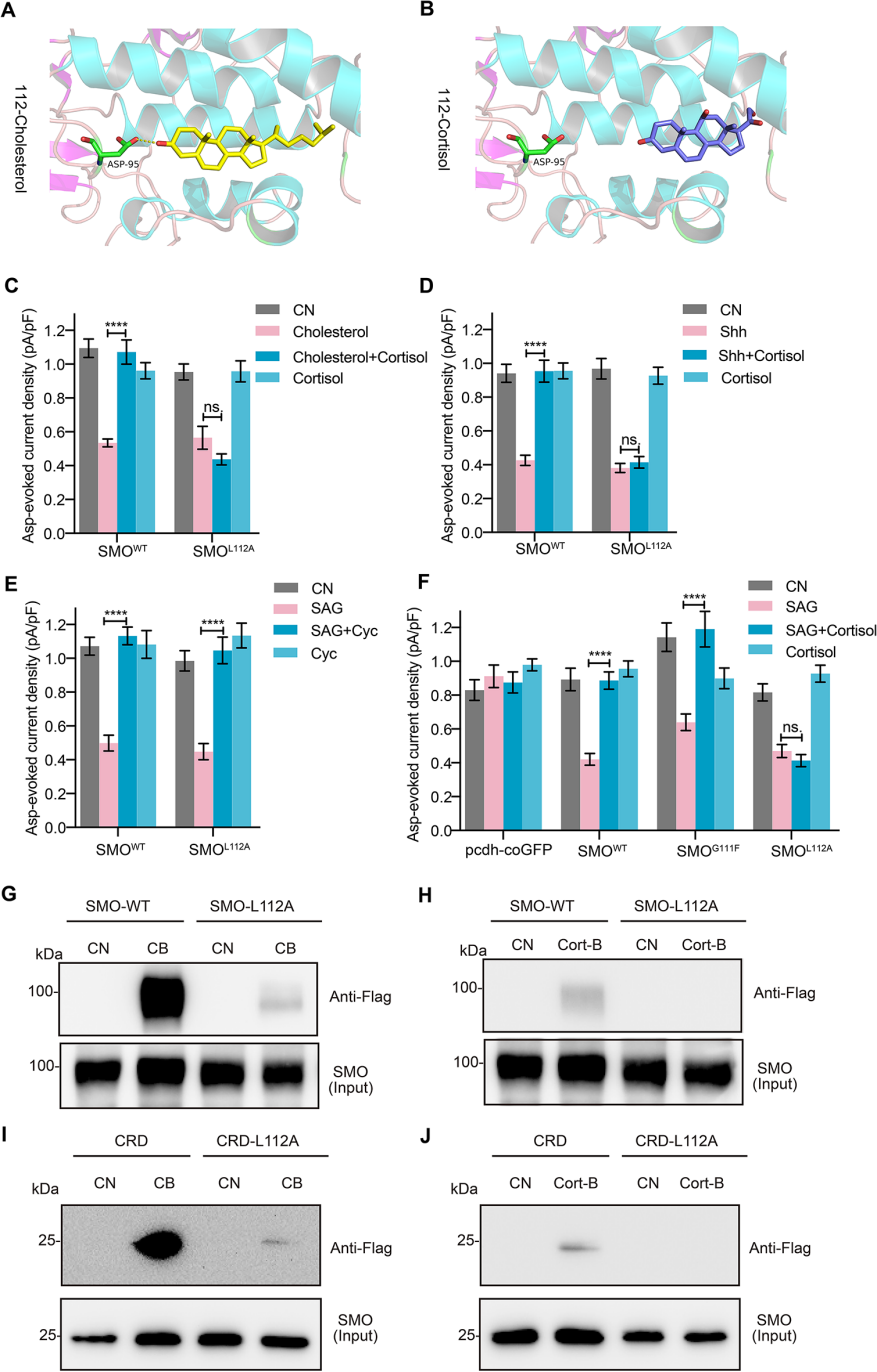
L112 of SMO was crucial for cortisol inhibition of SHH pathway. **A** and **B**, A stable pose for cholesterol (**A**) and cortisol (**B**) with CRD after A112 mutation of SMO predicted by molecular docking. **C**-**E**, Asp-evoked current density in HeLa cells transfected with SMO variants, WT or L112A incubated with cholesterol (**C**, *n* = 10–16) or Shh (**D**, *n* = 11–17) in the presence of cortisol. **E** and **F**, Asp-evoked current density in HeLa cells transfected with SMO variants, pcdh-coGFP (as a negative control), WT, G111F (as a SMO mutate negative control) and L112A in the presence of SAG and Cyc (**E**, *n* = 12–18) or cortisol (**F**, *n* = 10–17). **G** and **H**, Purified SMO WT or L112A mutate binding to biotinylated cholesterol (**G**) or biotinylated cortisol (**H**). **I** and **J**, Purified CRD of SMO WT or L112A mutate binding to biotinylated cholesterol (**I**) or biotinylated cortisol (**J**). Unless stated, CB, biotinylated cholesterol; Cort-B, biotinylated cortisol. All western blots performed three or more replicates. Data are represented as mean ± SEM. **** *p* < 0.0001; ns., no significance, *p* > 0.05. See also Supplementary Figs. 3 and 4

To identify the specific binding site(s) on the CRD responsible for cortisol binding, we simulated molecular docking of both wild type (WT) and mutant SMO with cholesterol and cortisol via Autodock. It has been reported that residues in SMO, such as L108, G111, and L112, are critical for sterol binding. Sterol binding occurs with the use of 20(S)-hydroxycholesterol (20(S)-OHC), an allosteric activator of SMO [15, 22, 45, 46, 65]. We mutated these sites and assessed the alterations in the docking energy with cholesterol or cortisol between the WT and the mutant SMO to estimate the binding capacity [42]. Simulating WT and L112A mutant SMO by cortisol or cholesterol resulted in an energy change from − 9.0 to − 8.1 kcal/mol or − 11.0 to − 10.9 kcal/mol, respectively (Supplementary Fig. 4A). These alterations suggested that the binding capacity variation of cortisol to L112A mutant SMO was nine times than that of cholesterol. In addition, L112A was not predicted by Autodock as the cortisol binding site (Fig. 3A and Supplementary Fig. 4B), but was still the cholesterol binding site (Fig. 3B and Supplementary Fig. 4B). Also, the L112A mutation did not affect the D95 hydrogen bond binding force to cholesterol (Fig. 3A), suggesting that the mutation had little effect on the binding of cholesterol to SMO. The G111F and L108A were not selected as specific binding sites for cortisol and cholesterol (Supplementary Fig. 4A).

To confirm that L112 is indeed crucial for cortisol to act on SMO in cells, we expressed the WT and L112A mutant forms of SMO in HeLa cells, a cell line expressing EAAC1 and SMO (www.proteinatlas.org) that is not responsive to SAG (Supplementary Fig. 4C-E). The Asp-evoked current was not affected by the expression of WT or mutant SMO in these cells (Supplementary Fig. 4F). Furthermore, mifepristone or eplerenone did not change the effects of cortisol on the current density (Supplementary Fig. 4G), suggesting that the inhibitory effects of cortisol on SMO were independent of GRs and MRs.

Cortisol recovered the cholesterol- (Fig. 3C and Supplementary Fig. 4H) and the Shh-mediated reduction (Fig. 3D and Supplementary Fig. 4I) in the Asp-evoked current in the HeLa cells expressing WT SMO. In contrast, cortisol did not change the Asp-evoked current in the cells expressing the L112A mutant SMO. In addition, corticosterone, another glucocorticoid, restored the Asp-evoked current that was reduced by cholesterol (Supplementary Fig. 4J) or SAG (Supplementary Fig. 4K) in the WT SMO cells. The corticosterone inhibition was not observed in the L112A mutant SMO cells. Previous studies have shown that cyclopamine acts on 7TMD of SMO [10, 62]. The cyclopamine effects on the Asp-evoked current were not changed in the HeLa cells expressing L112A SMO (Fig. 3E). Expression of G111F SMO did not alter the inhibitory effects of cortisol on Asp-evoked current induced by SAG (Fig. 3F). These results suggested that the cortisol inhibited the cholesterol binding to CRD, but not to 7TMD, and L112, but not G111, was the specific binding site for cortisol to SMO. To further examine that L112 of SMO is indeed important for cortisol binding, we performed the binding experiments. The CB remained binding to the purified WT and L112A SMO (Fig. 3G). In contrast, the Cort-B did bind to the WT SMO, but did not bind to the L112A SMO (Fig. 3H). Moreover, CB did bind to the L112A mutated CRD fragments (Fig. 3I), whereas Cort-B did not bind to the L112A mutated CRD fragments (Fig. 3J). All of these results suggested that L112 of SMO was necessary for cortisol to bind to the CRD of SMO and inhibit the activation of SMO.

### Removal of cortisol inhibition on SMO promoted cell proliferation resulting in immature lung development

**Fig. 4:**
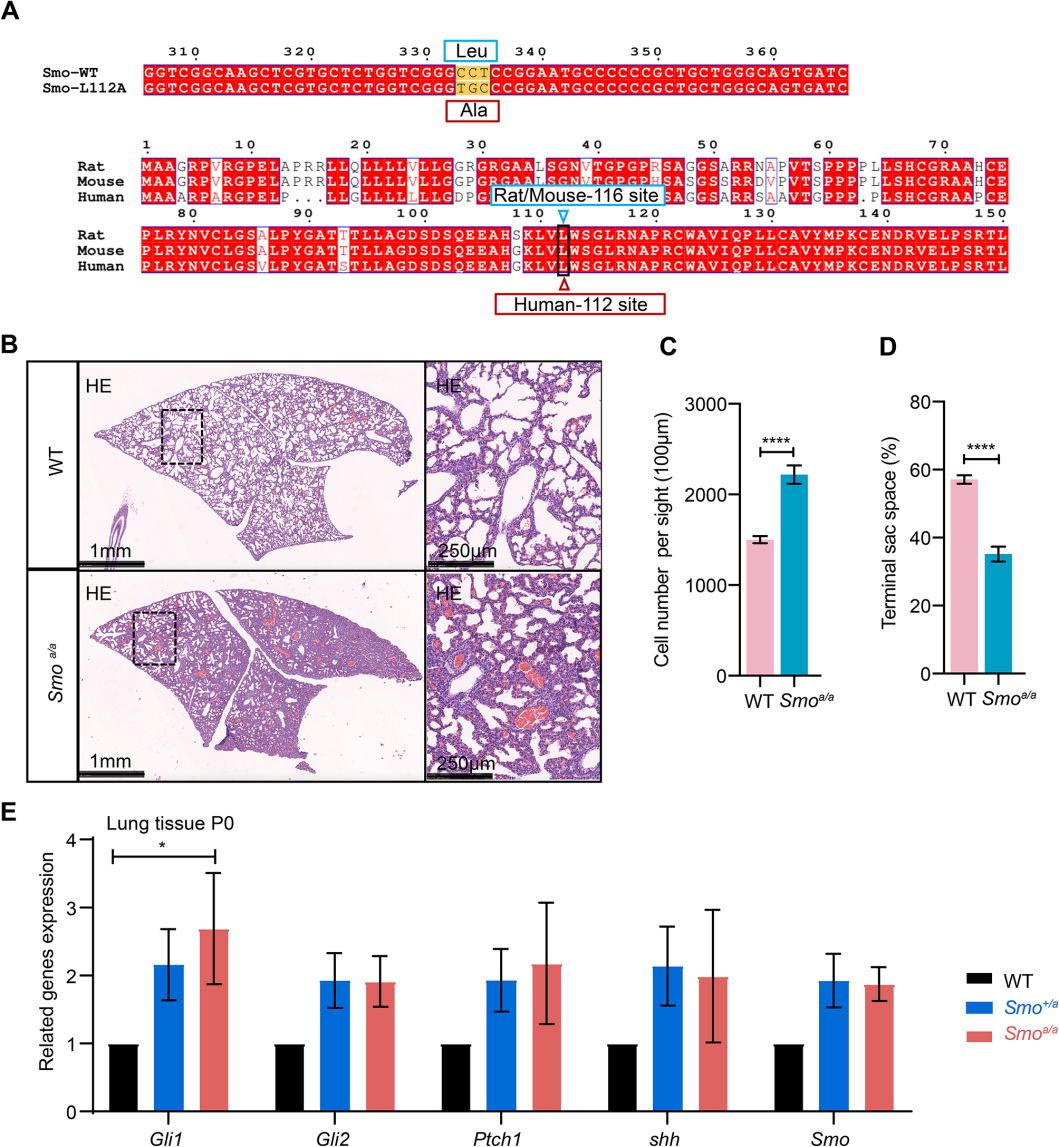
Loss of cortisol inhibition to SMO led to immature lung development. **A**, The sequence alignment of SMO orthologues in different species. **B**, Typical images of H&E-stained lung sections from newborn WT and *Smo*^*a/a*^ mice. **C** and **D**, Statistics of cell number of lung slices from newborn WT (*n* = 23 from five mice) and *Smo*^*a/a*^ (*n* = 24 from four mice) mice (**C**). Statistics of terminal sac space in lung slices from newborn WT (*n* = 40 from eight mice) and *Smo*^*a/a*^ (*n* = 29 from six mice) mice (**D**). The cell number per sight (100µm^2^) was used to indicate the average cell numbers quantification of slices. Each section counted five fields from top, bottom, left, right and middle. **E**, The SHH signaling expression of lung tissue at P0. Data are represented as mean ± SEM. * *p* < 0.01; **** *p* < 0.0001; ns., no significance, *p* > 0.05. See also Supplementary Fig. 5

To explore the effect of cortisol action on the SHH pathway on neonatal lung development, we generated a genetic mouse. Since the amino acid 116 of SMO protein in mice corresponds to the 112 site in human (Fig. 4A), we mutated leucine 116 of SMO to alanine 116 (L116A, *Smo*^*a/a*^) by the CRISPR-Cas9 (Supplementary Fig. 5A). We first observed the morphology and appearance of the transgenic mice. These mice had dark red skin and a reduced breathing rate (Supplementary Fig. 5B and Supplementary Movie 1). There was no obvious difference in anatomical observations between WT and *Smo*^*a/a*^ mice, including body shape, weight (Supplementary Fig. 5B, C), and organs such as the brain, lung, heart, kidney, and liver (Supplementary Fig. 5D-H). Histological examinations revealed no obvious abnormalities in the brain, heart, kidney, or liver of *Smo*^*a/a*^ mice. Lung sections exhibited markedly thickened interstitial parts and greatly reduced alveolar cavities in *Smo*^*a/a*^ mice compared to those in WT mice (Fig. 4B). The statistical analysis showed that in *Smo*^*a/a*^ mice, the cell number in the visible sights was markedly increased (Fig. 4C) and the terminal sac spaces in the lungs were greatly reduced (Fig. 4D). The *Smo*^*a/a*^ mice died shortly after birth. There was no difference observed in Masson trichrome staining between WT and *Smo*^*a/a*^ mice (Supplementary Fig. 5I), suggesting that the thickened interstitial parts were not associated with collagenous fibroplasia. Next, we examined the gene expression related to SHH pathway of lung tissue from homozygous mice at postnatal 0 day (P0). It has been reported that cortisol increases in the third trimester of pregnancy [67]. It is therefore possible that cortisol inhibition of SHH pathway affects lung development and maturation. Indeed, there was a significant increase of *Gli1* expression at P0 (Fig. 4E), indicating that the removal of cortisol inhibition to SMO kept the high activation of SHH pathway in *Smo*^*a/a*^ mice. Meanwhile, the expression of *shh* and *ptch1* had no change (Fig. 4E), indicating that cortisol did no effect on the upstream of SHH pathway.

**Fig. 5:**
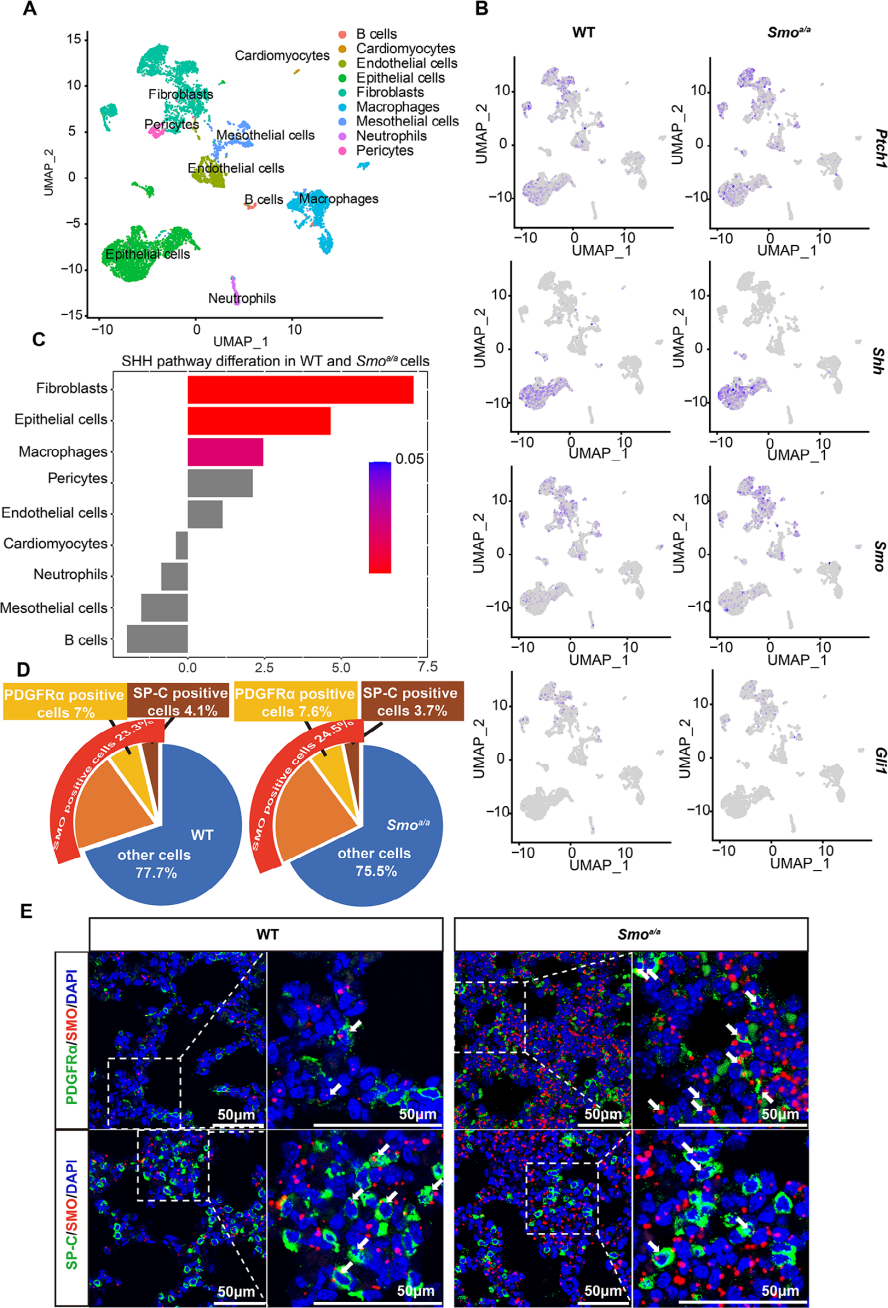
SHH pathway in newborn WT and *Smo*^*a/a*^ mice. **A**, UMAP plot of cell clusters from two groups merged of lung tissues. **B**, UMAP plot of lung tissue cells displaying marker gene expression. **C**, SHH Pathway Enrichment Analysis of variation in the fibroblasts and epithelial cells clusters between WT and *Smo*^*a/a*^ mice. **D**, statistical results of main function of cell clusters. **E**, Fluorescence in-situ hybridization (FISH) staining of lung from WT and *Smo*^*a/a*^ mice at P0 with indicated antibodies. Scale bar = 50 μm. The white arrows meaning the co-labeled cells with SMO probe and PDGFRα or SP-C antibodies. See also Supplementary Fig. 5

To find more evidence of the cortisol influence on lung development, we performed single-cell RNA sequencing (scRNA-seq) on lung tissue from three neonatal wild mice and three neonatal *Smo*^*a/a*^ mice. The cells from lung tissues were grouped into nine clusters (Fig. 5A), and the *Ptch1*,* Shh*,* Smo*,* Gli1* genes, which are the main elements of the SHH pathway, were mainly expressed in fibroblasts and epithelial cells (Fig. 5B). SHH Pathway Enrichment Analysis also showed great variation in the fibroblasts and epithelial cells clusters between WT and *Smo*^*a/a*^ mice (Fig. 5C), and the expression of SHH pathway elements was higher in *Smo*^*a/a*^ mice than WT (Supplementary Fig. 6A). As the thickened interstitial parts and greatly reduced alveolar cavities, the cell composition of the lung tissues analysis showed that the number of SMO-positive cells was highly increased from 23.3 to 24.5% in *Smo*^*a/a*^ mice, comparing with WT mice (Fig. 5D). And the proportion of PDGFRα-positive cells, a kind of fibroblasts capable of growth, was increased from 7 to 7.6% in *Smo*^*a/a*^ mice compared to WT mice, while the SP-C positive cells, which secretes prosurfactant protein C, were reduced from 4.1 to 3.7% (Fig. 5D).

Then, we did fluorescence in situ hybridization (FISH) to examine the alterations, using probe-labeled SMO, and antibodies against PDGFRα, or prosurfactant protein C to label fibroblasts, or surfactant proteins. The SMO-positive cell numbers were greatly increased in *Smo*^*a/a*^ mice compared to WT mice (Fig. 5E and Supplementary Fig. 6B). In *Smo*^*a/a*^ mice, PDGFRα-positive cells among SMO-positive cells were almost doubled and surfactant protein-positive cells in the interstitial area were reduced by about 40% (Fig. 5E, Supplementary Fig. 6C, D). These observations suggested that the thickened interstitial parts occurred owing to an increase in the fibroblasts. The decreased surfactant proteins in *Smo*^*a/a*^ mice supported the idea that glucocorticoids can promote the increase of surfactant proteins in preterm infants [21, 34, 38].

**Fig. 6:**
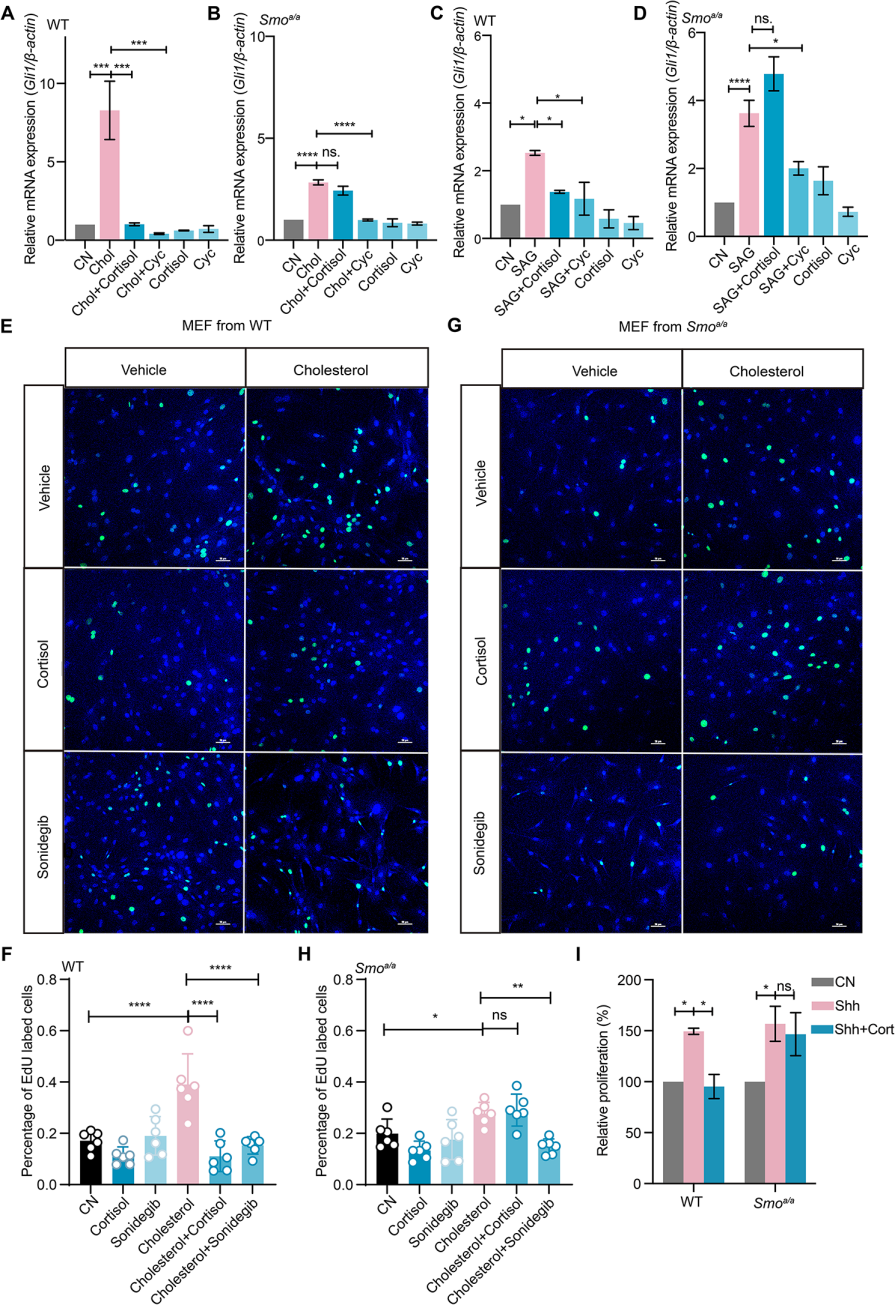
Cell proliferation of MEFs from WT and *Smo*^*a/a*^ mice. **A-D**, The *Gli1* expression in mouse embryonic fibroblasts (MEFs). **A** and **B**, Relative mRNA expression of *Gli1* in MEFs isolated from WT (**A**, *n* = 3) and *Smo*^*a/a*^ (**B**, *n* = 6) mice incubated with cholesterol in the presence of cortisol or Cyc. **C** and **D**, Relative mRNA expression of *Gli1* in WT or *Smo*^*a/a*^ MEFs incubated with SAG (**C**, *n* = 3 and **D**, *n* = 6). **E-H**, The proliferated MEFs isolated from WT (**E** and **F**) and *Smo*^*a/a*^ (**G** and **H**) mice labeled by EdU incubated with cholesterol in the presence of cortisol or SMO antagonist, Sonidegib. **I**,The proliferation of MEFs isolated from WT and *Smo*^*a/a*^ mice incubated with shh and cortisol for 24 h, *n* = 3.Unless stated, WT, wild type; Chol, Cholesterol; Cort, cortisol; Cyc, cyclopamine. Data are represented as mean ± SEM. ** *p* < 0.01, **** *p* < 0.0001;ns., no significance, *p* > 0.05. See also Supplementary Fig. 6

We also isolated the mouse embryonic fibroblasts (MEFs) from WT and *Smo*^*a/a*^ mice and examined the effects cholesterol on SHH pathway in the MEFs. The *Gli1* expression in WT MEFs was induced by cholesterol (Fig. 6A) in a cortisol-sensitive manner. In contrast, cortisol did not change the elevated *Gli1* expression in *Smo*^*a/a*^ MEFs (Fig. 6B). Similarly, the inhibitory effect of cortisol on SAG-induced *Gli1* expression was not found in *Smo*^*a/a*^ MEFs (Fig. 6C, D and Supplementary Fig. 6E-H). We found that the elevated *Gli1* expression by cholesterol was decreased from 8-fold to 3-fold in *Smo*^*a/a*^ (Fig. 6A, B), while there were no significant changes in *Gli1* expression in response to SAG (Fig. 6C, D), indicating that the L116 mutation of SMO did not affect the SAG action. It has been reported that SAG activates the SHH pathway by binding to the 7TMD of SMO while cholesterol by binding to the CRD and 7TMD [6, 22, 23]. Therefore, it was possible that the L116A mutation in the CRD of SMO partially blocked cholesterol activation of the SHH pathway. Next, we assayed the cell proliferation of MEFs treated with cholesterol. We first examined cell proliferation by EdU proliferation assay. The cholesterol promoted the cell proliferation of both WT and *Smo*^*a/a*^ MEFs. The proliferation was inhibited by sonidegib (SMO antagonist known to bind to 7TMD of SMO) and cortisol (Fig. 6E, F) in WT MEFs. The inhibitory of cortisol was not found in *Smo*^*a/a*^ MEFs (Fig. 6G, H), but sonidegib remained inhibiting the proliferation of *Smo*^*a/a*^ MEFs. To further confirm the effect of cortisol on cell proliferation, CCK8 assay was performed to detect cell proliferation. The proliferation of WT MEFs induced by Shh was greatly suppressed in the presence of cortisol; however, this inhibition was not observed in *Smo*^*a/a*^ MEFs (Fig. 6I). These results provided evidences that cortisol could not inhibit SMO activity in *Smo*^*a/a*^ mice, supporting the idea that the activated SHH pathway promotes the cell proliferation, while cortisol inhibits the cholesterol-induced cell proliferation, and the removal of cortisol inhibition to SMO may be the main cause of cell over-proliferation in *Smo*^*a/a*^ mice.

All of the results exhibited by *Smo*^*a/a*^ mice were thus consistent with an explanation that loss of cortisol inhibition on SMO leads to maintained stimulation of SHH pathway, which might affect normal lung development in neonatal mice, with changes similar to those of respiratory distress disorder.

## Discussion

The main finding of the current study is that cortisol inhibited SMO-mediated fibroblasts proliferation to regulate neonatal lung development in mice. Mechanismly, cortisol inhibited the activated SHH pathway by directly binding to the CRD of SMO and loss function of cortisol on SMO caused over-proliferated fibroblasts resulting in immature lung development. Several experimental results in this study supported this conclusion. First, when SMO was activated, cortisol directly bound to the CRD and competed with cholesterol to suppress Shh/SMO signaling. Second, the L112A SMO (site 116 in mice) did not bind to cortisol, and the removal of cortisol inhibition caused the activated SHH pathway and decreased surfactant proteins in *Smo*^*a/a*^ mice. Finally, eliminating the inhibition of cortisol to SMO resulted in over-proliferated fibroblasts and immature lung development. Our results were thus consistent with the model (Supplementary Fig. 6I) in which cholesterol stimulated SMO by binding to the CRD, while cortisol competed with the cholesterol in the CRD to inhibit the activation of SMO and controlled lung development.

An important implication is that cortisol modulation of SMO activation was likely crucial for lung maturation in mice. In development, Shh is released from epithelial cells to promote the proliferation of fibroblasts and epithelial cells in different tissues [11, 16, 30, 40, 58], and it is expressed in respiratory epithelial cells in complex and variable patterns at the onset of lung growth at day E10 [25, 56]. *Shh* knock-out mice show simple pulmonary hypoplasia, the decrease in the number of lung epithelial and mesenchymal cells [49]. Inversely, *Shh* overexpression driven by *SP-C* promoterresults in an increase in mesenchymal cell proliferation and an absence of typical alveoli, and the transgenic mice die soon after birth [2]. In humans, lung dysplasia is also one of the most notable causes of early neonatal morbidity and mortality [17, 36]. Administration of glucocorticoids greatly reduces the incidence of dysplasia for newborns [14, 15, 47, 48, 51, 55]. It has been reported that the glucocorticoid receptor (GR) agonist also binds to the Hedgehog pathway receptor, Smoothened, to inhibit the SHH pathway, such as dexamethasone, fluorometholone [9, 32, 61, 63]. In addition, the mutation in *Dhcr7*, a key gene for cholesterol synthesis, causes late gestational lung hypoplasia in mice [66]. Mice with a loss function of hedgehog interacting protein, an antagonist of SHH pathway, die a few hours after birth due to respiratory failure [13]. The homozygous *Smo*^*D99N/D99N*^ knock in mice are embryonic lethal with severe cardiac defects [65], and here *Smo*^*a/a*^ mice died after birth with lung defects, all suggested that the L116A mutation did affect the cortisol inhibition of SMO, leading to increase in the proliferation of interstitial fibroblasts. The increase of cortisol during pregnancy occurs during 3rd trimester, a critical period for lung development [67]. Thus, the balance of the stimulation and inhibition of Shh/SMO signaling supports the development of the lung during which cortisol acts as an endogenous antagonist that competes with cholesterol to modulate SMO activity and lung development.

SMO, a crucial signal transducer of the HH pathway, is controlled by the signaling condition switch [23, 57]. The CRD and 7TMD in SMO are the two key regulatory sites. The CRD has the sterols binding pocket and can swap the stimulation of SMO by recruiting and binding to cholesterol [6, 22, 23], whereas the 7TMD can function as a longitudinal channel to transport the sterol to the CRD and stimulate SMO [23, 52]. Now, we showed here that cortisol inhibited SMO via the CRD (Fig. 3C-F), but not via the 7TMD. Chemical SMO antagonists inhibit SMO by preventing the outward movement of cholesterol via the 7TMD [52, 57]. This is different from the inhibition of SMO by blocking cholesterol transporting to the CRD. In this context, cortisol inhibition of SMO might be as a result of endocrine regulations, a novel mechanism to reveal the endogenous regulation of Shh/SMO signaling under physiological or pathological conditions.

Another important finding of the current study is that cholesterol and cortisol had different binding affinities with the CRD. In the simulation analysis, the minimum binding energy to SMO for cholesterol or cortisol in the CRD was − 11.0 kcal/mol or − 9.0 kcal/mol, respectively. Additionally, there were 17 or 10 amino acids, estimated for the binding of cholesterol or cortisol, respectively, to the CRD. These assessments provided the structural basis to explain why cholesterol or cortisol had different affinities to the CRD. Moreover, the mutation of L112A in SMO resulted in a nine-fold difference in the binding capacity between cholesterol and cortisol [42]. Similar to these simulating results, cortisol required a higher incubating concentration to bind to SMO than cholesterol in the binding assay (Fig. 2E, F). One µM cortisol could inhibit the cholesterol function on SHH pathway different from the 10–100 µM in the cell-free binding experiments. It should be noted that L116A SMO exhibited reduced cholesterol binding, but this reduction obviously did not affect SHH signaling activation. It has been previously reported L116A mutants don’t show a strong binding to 20(S)-OHC beads in the in vitro assay, but respond to 20(S)-OHC when transfected into *Smo*^*−/−*^ cells [46]. This discrepancy in binding and in functional assays may reflect the fact that the signaling in intact cells is more sensitive than the binding with purified proteins. In Supplementary Fig. 2I, we showed 5 µM cholesterol that promoted the Cort-B binding to purified SMO. This observation was consistent with an explanation that the binding of the low-dose cholesterol to SMO induced the structural changes in the SMO, resulting in a momentary increase of cortisol binding. As the concentration of cholesterol increases, it produced a competitive inhibitory effect on cortisol that gradually reduced the binding of cortisol to SMO. These observations suggested that cortisol and cholesterol competed in the same pocket in the CRD and the 112 site in CRD of SMO was really important for cortisol binding. Therefore, the CRD of SMO acted as the ultimate activation trigger to turn on the SHH pathway, while cortisol acted as the endogenous modulator of SMO via the CRD.

The limitation of this study is that the inhibitory effect of cortisol on the SHH pathway and its binding to the CRD domain of SMO have been tested only at the molecular, cellular, and animal levels. Current evidence is insufficient to elaborate the binding type between cortisol and SMO or the form of the binding force. In addition, the postnatal death of homozygous mice is associated with the effects of cortisol on the inhibition of the SHH pathway. More experiments are needed to further clarify these questions.

A possible implication of the current investigation is that the cortisol inhibition of SMO activation provide a novel therapeutic opportunity for Shh/SMO inhibitors to be used for the management of different diseases. Different synthetic forms of cortisol are currently used to treat a variety of diseases, including pneumonia and asthma [20, 26, 43, 53, 54]. It is generally believed that cortisol works by controlling inflammation [8]. Since the SHH pathway is activated in many diseases [29], it is possible that cortisol affects the progress of these diseases by modulating the SHH pathway. Thus, developing drugs targeting CRD binding pocket of SMO might specifically inhibit the SHH pathway and avoided the side effects of glucocorticoids in the treatment of these diseases.

## Conclusions

The cortisol inhibition to SMO was important for the regulation of fibroblast proliferation in the neonatal lung. Demonstration of cortisol modulation of SMO enhanced our understanding of the regulation of SHH pathway and the diverse roles of cortisol. The balance of stimulation and inhibition of Shh/SMO signaling supported the development of the lung. The aberrant cortisol levels in late trimester of pregnancy decreased the inhibition of SHH pathway, likely the main cause of NRDS.

## Electronic supplementary material

Below is the link to the electronic supplementary material.

Supplementary Material 1

Supplementary Material 2

Supplementary Material 3

Supplementary Material 4

## Data Availability

Data is provided within the manuscript or supplementary information files.

## Abbreviations

NRDS: Neonatal respiratory distress syndrome
SMO: Smoothened
Shh: Sonic hedgehog
HH: Hedgehog
CRD: Cysteine-rich domain
ΔCRD: CRD-free domain
7TMD: Seven-transmembrane domain
SAG: Smoothened agonist
GCs: Glucocorticoids
Asp: Aspartate
GRs: Glucocorticoid receptors
MRs: Mineralocorticoid receptors
CB: Biotinylated cholesterol
Cort-B: Biotinylated cortisol
WT: Wild type
20(S)-OHC: 20(S)-hydroxycholesterol
scRNA-seq: Single-cell RNA sequencing
FISH: Fluorescence in situ hybridization
MEFs: Mouse embryonic fibroblasts
