# Supplementary material for: Cortisol regulates neonatal lung development via Smoothened
Source: Respir Res. 2025 Jan 18;26:27. doi: 10.1186/s12931-025-03104-0 (PMC11743026; doi:10.1186/s12931-025-03104-0)
Supplement: Supplementary file 2 — Supplementary Material 2 [file 12931_2025_3104_MOESM2_ESM.pptx]

## Slide 1
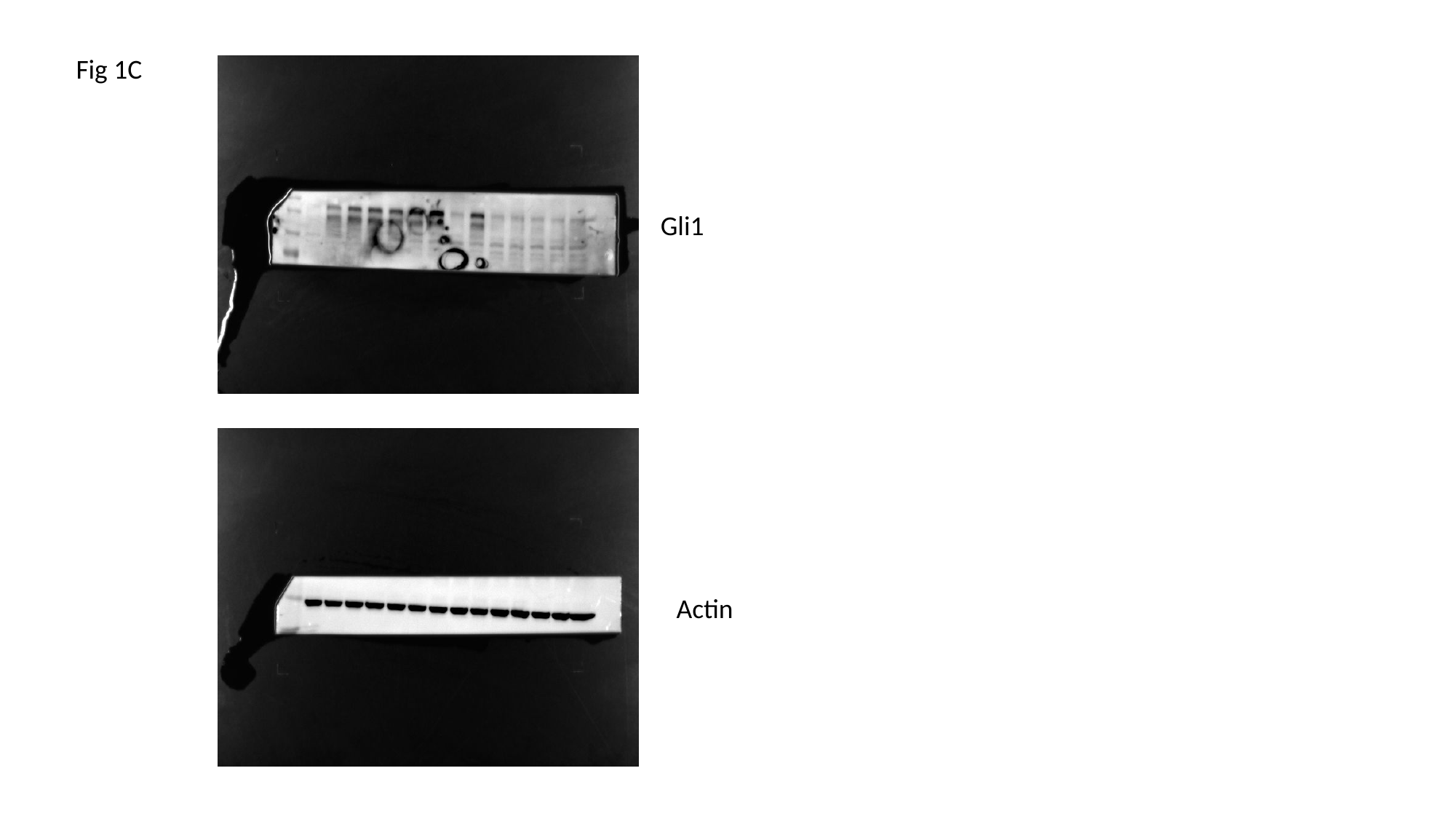

Fig 1C
Gli1
Actin

## Slide 2
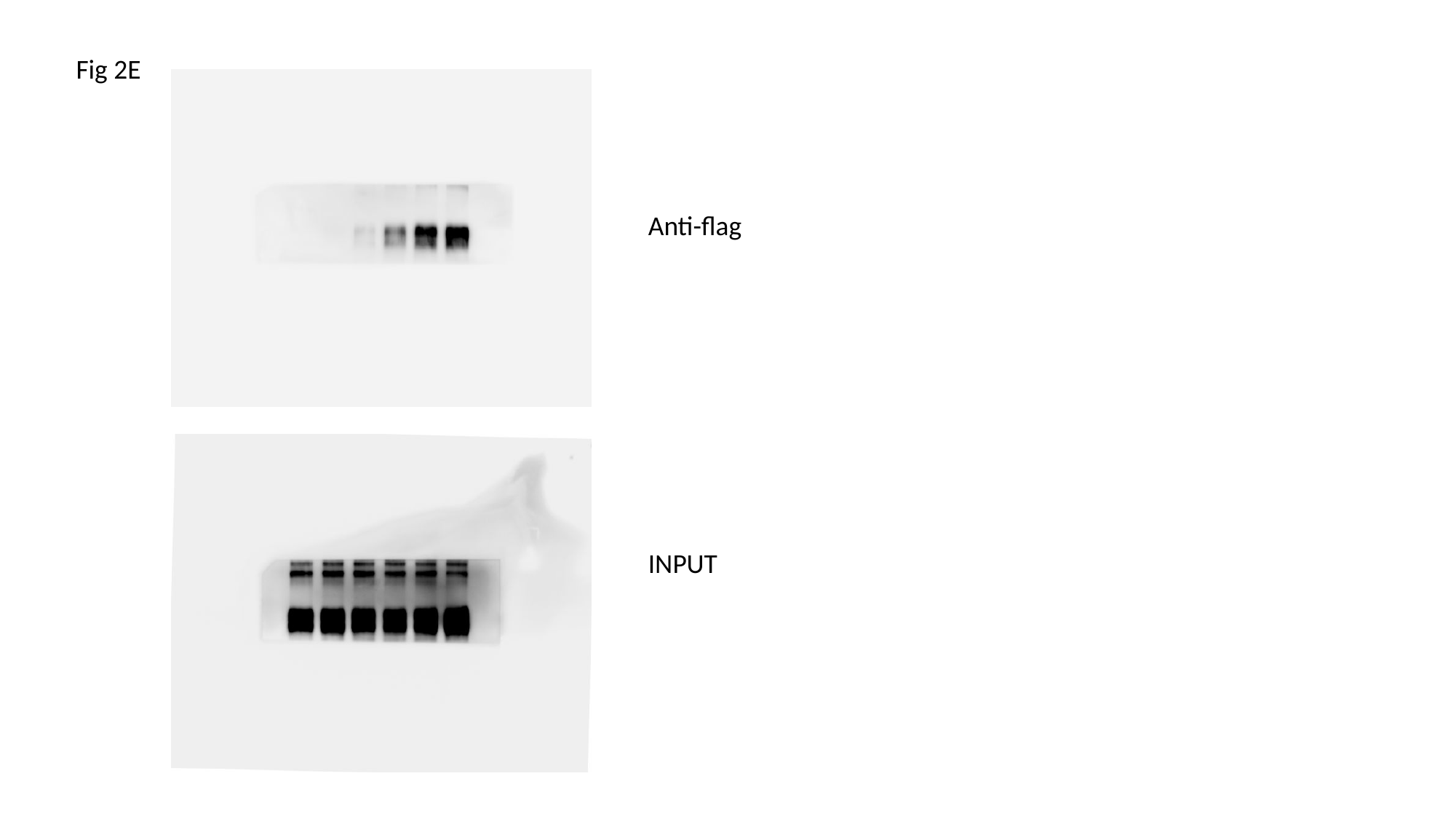

Fig 2E
Anti-flag
INPUT

## Slide 3
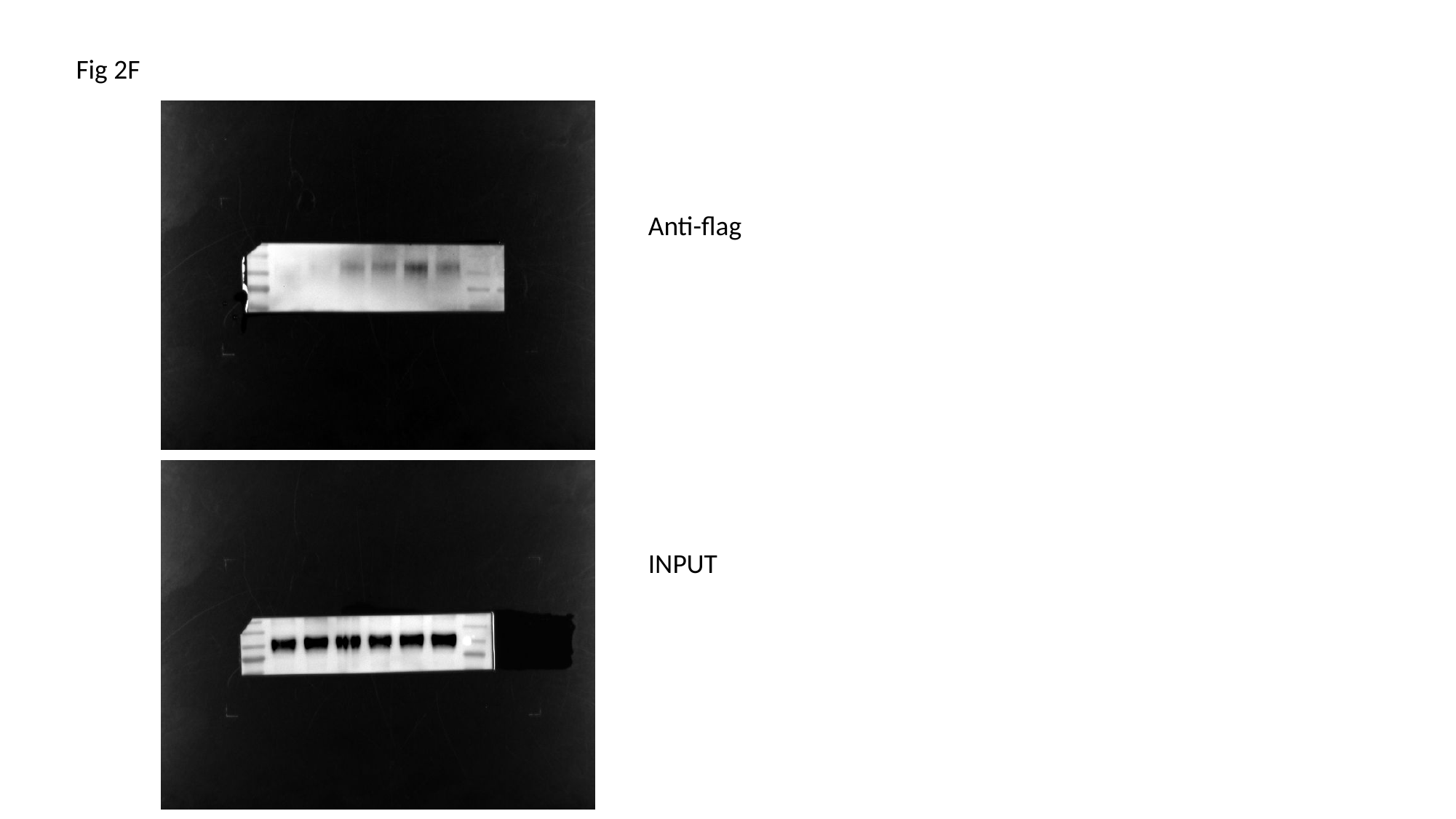

Fig 2F
Anti-flag
INPUT

## Slide 4
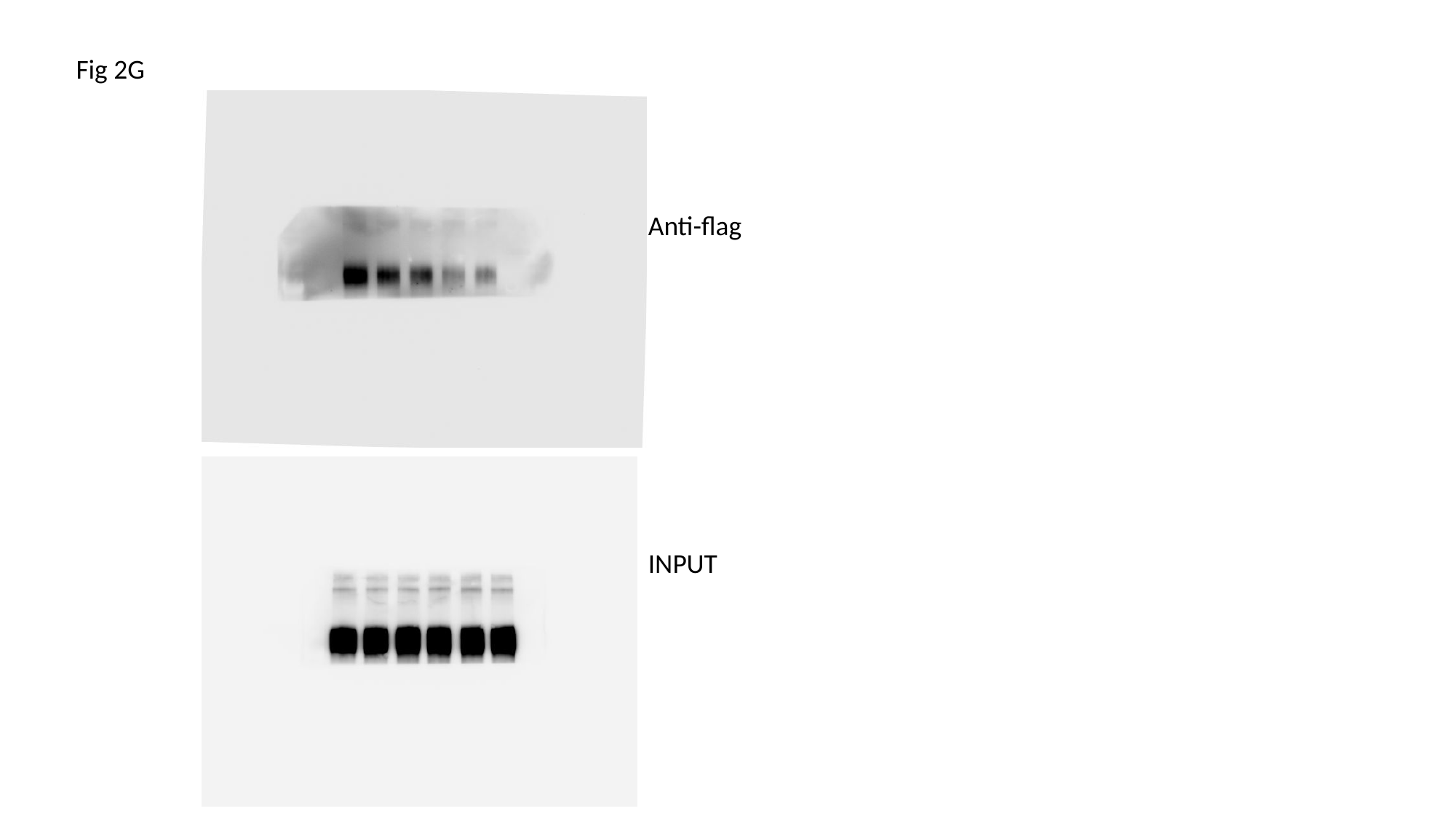

Fig 2G
Anti-flag
INPUT

## Slide 5
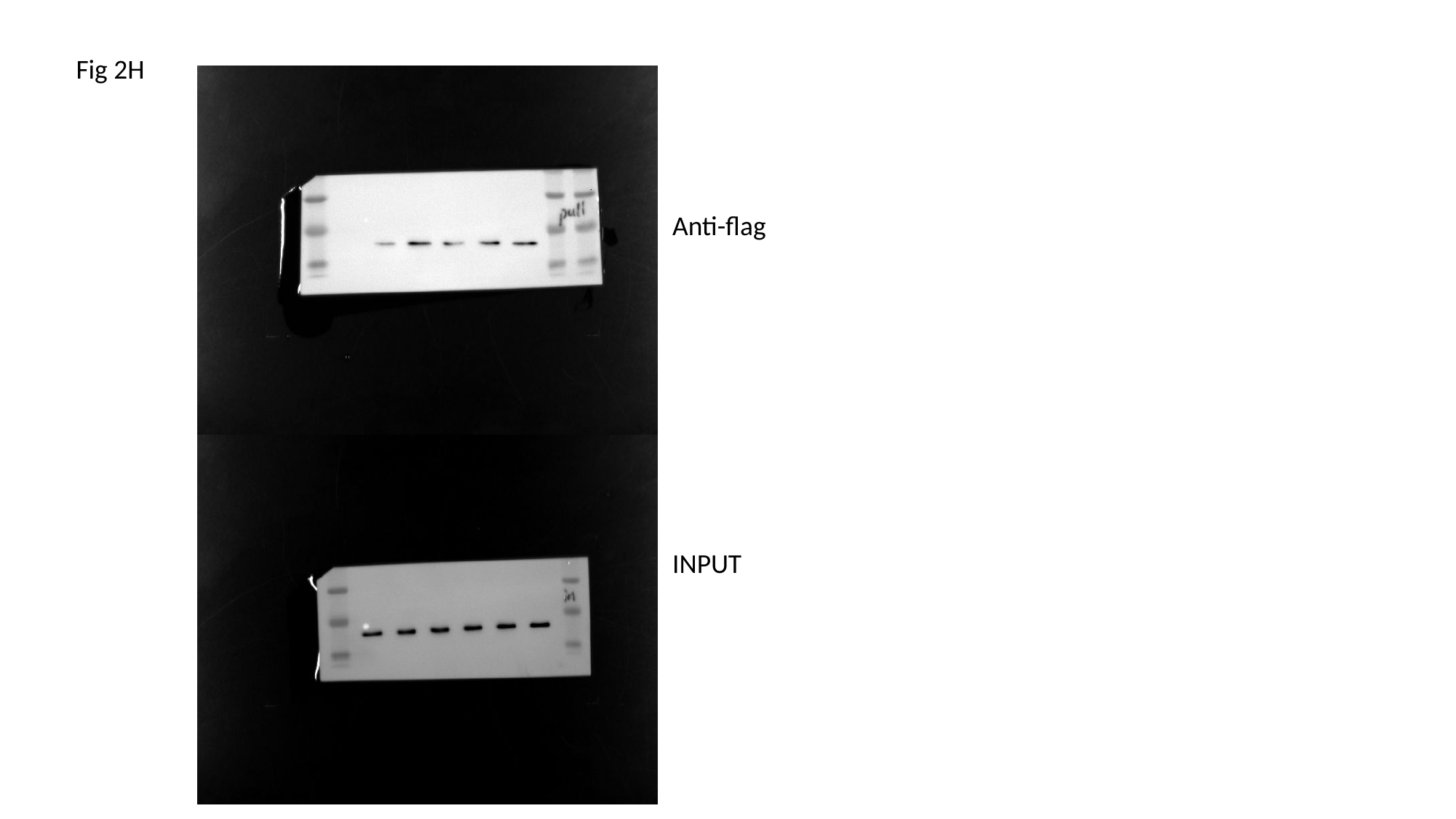

Fig 2H
Anti-flag
INPUT

## Slide 6
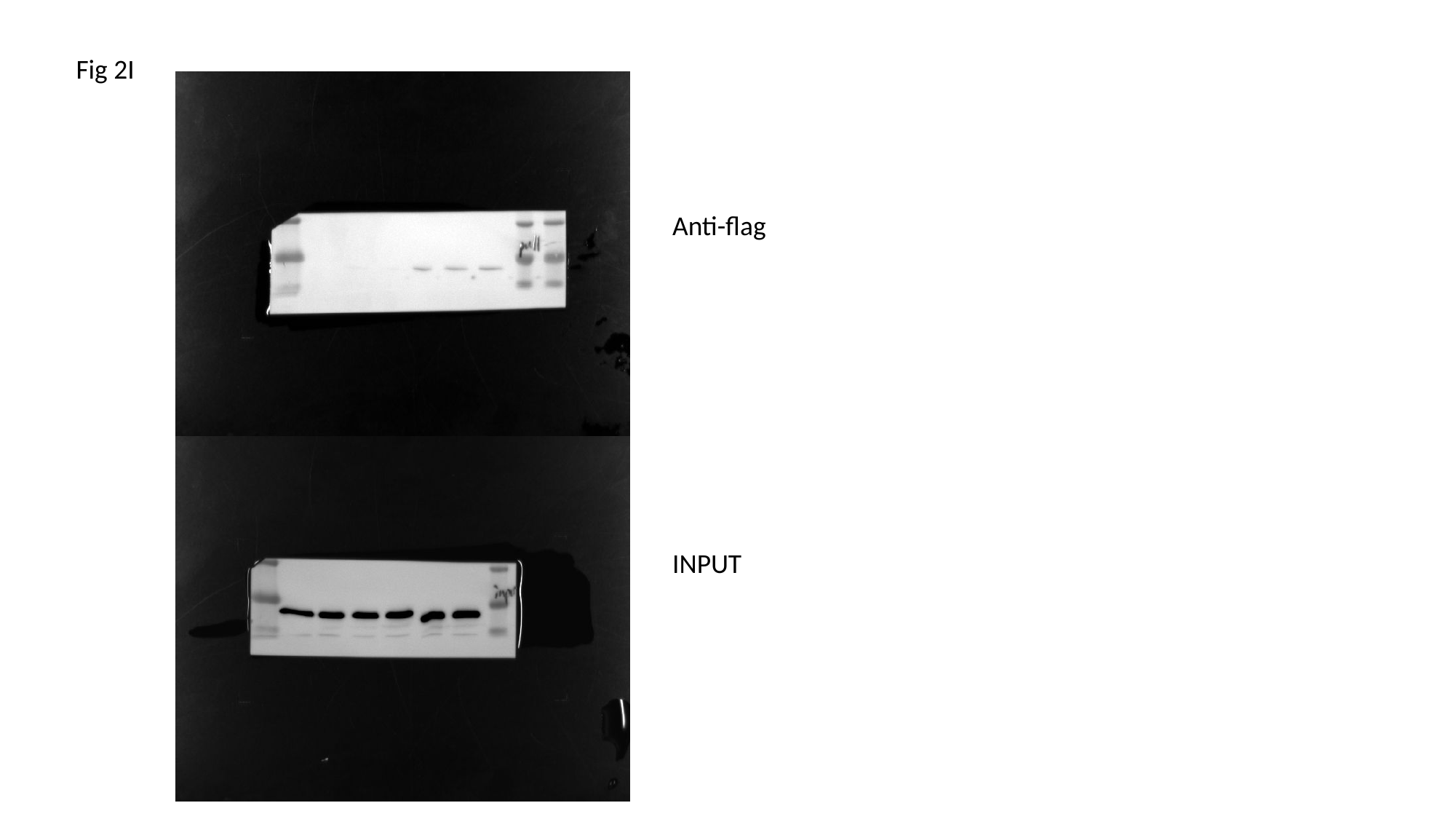

Fig 2I
Anti-flag
INPUT

## Slide 7
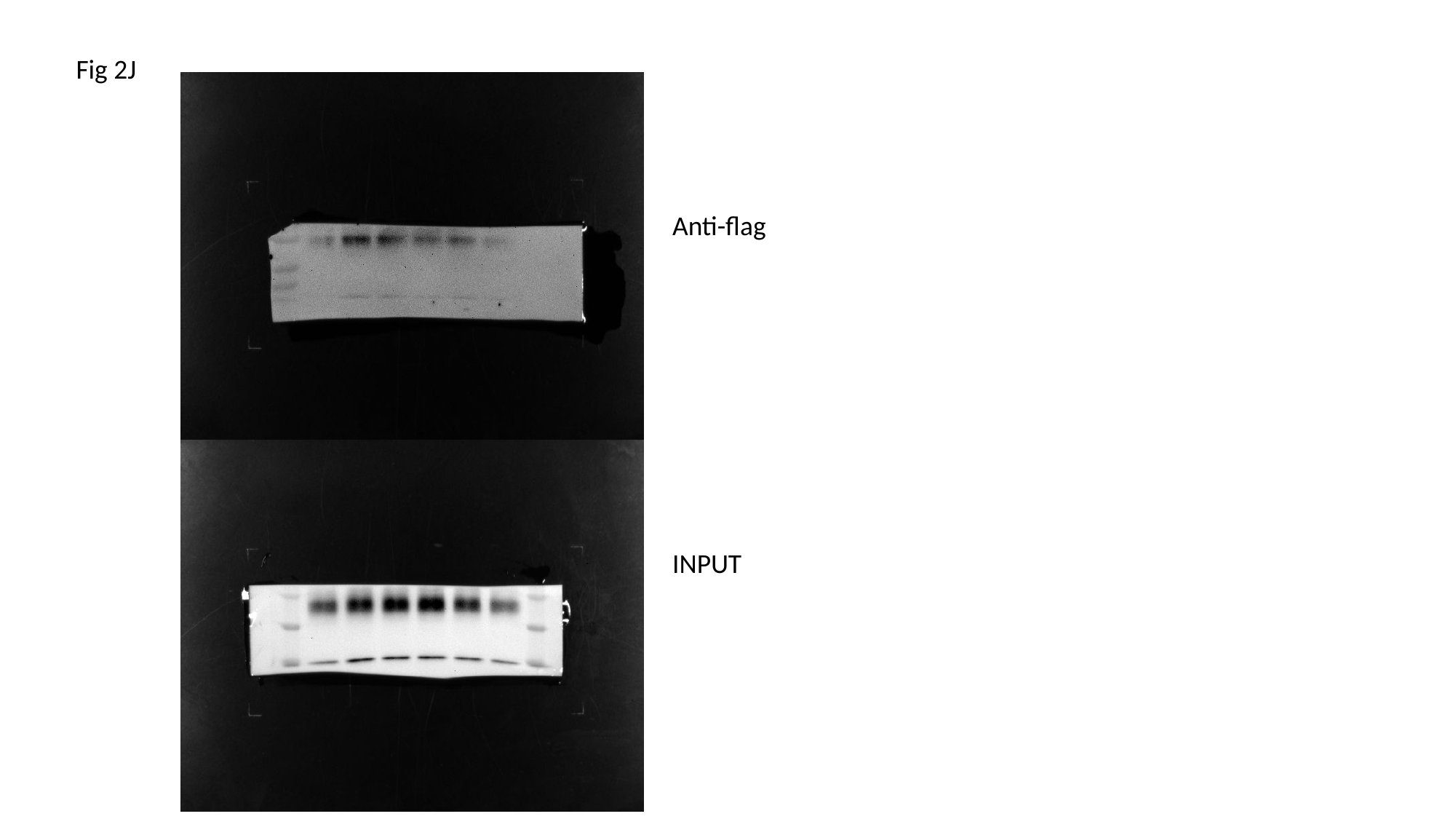

Fig 2J
Anti-flag
INPUT

## Slide 8
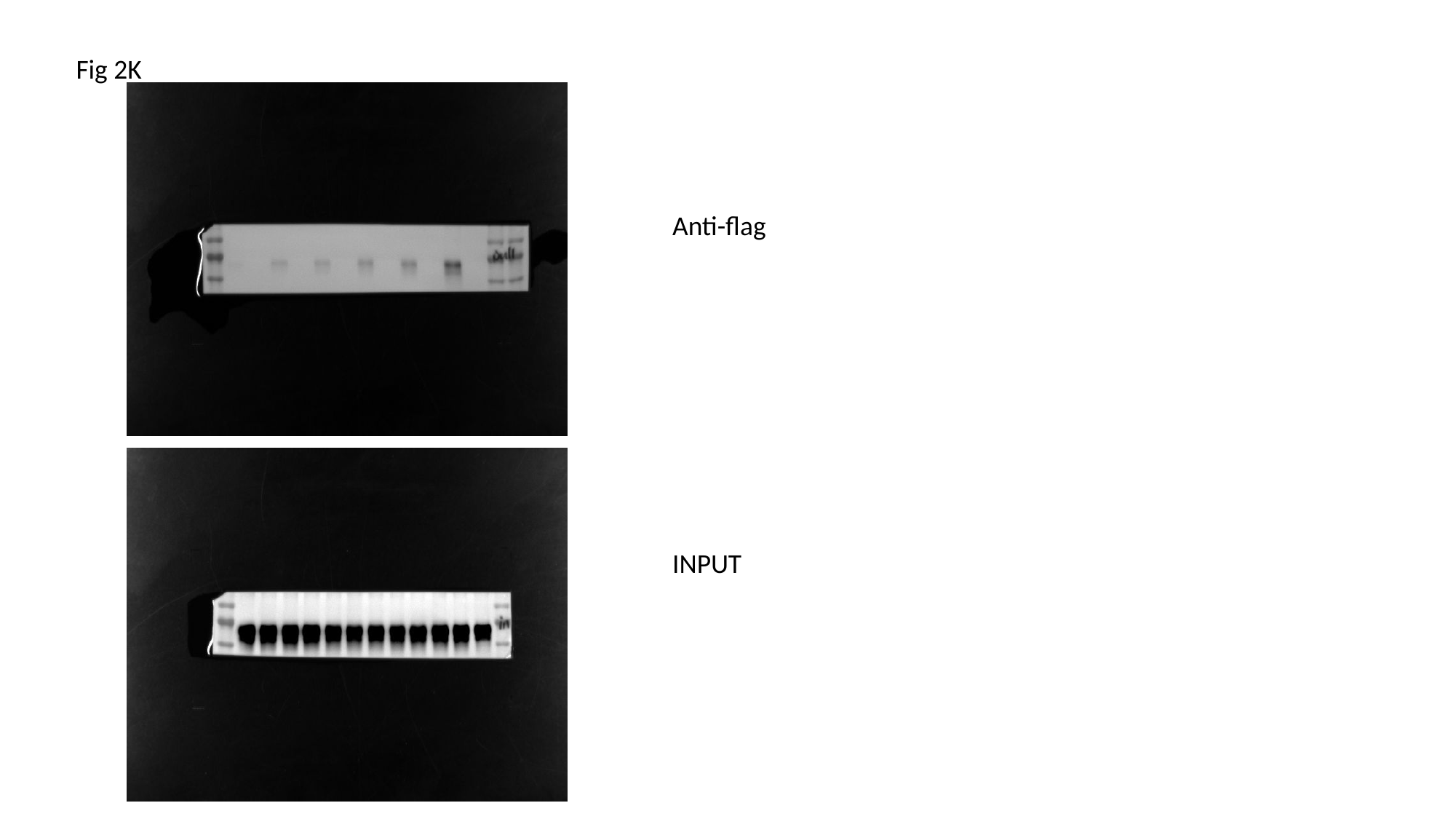

Fig 2K
Anti-flag
INPUT

## Slide 9
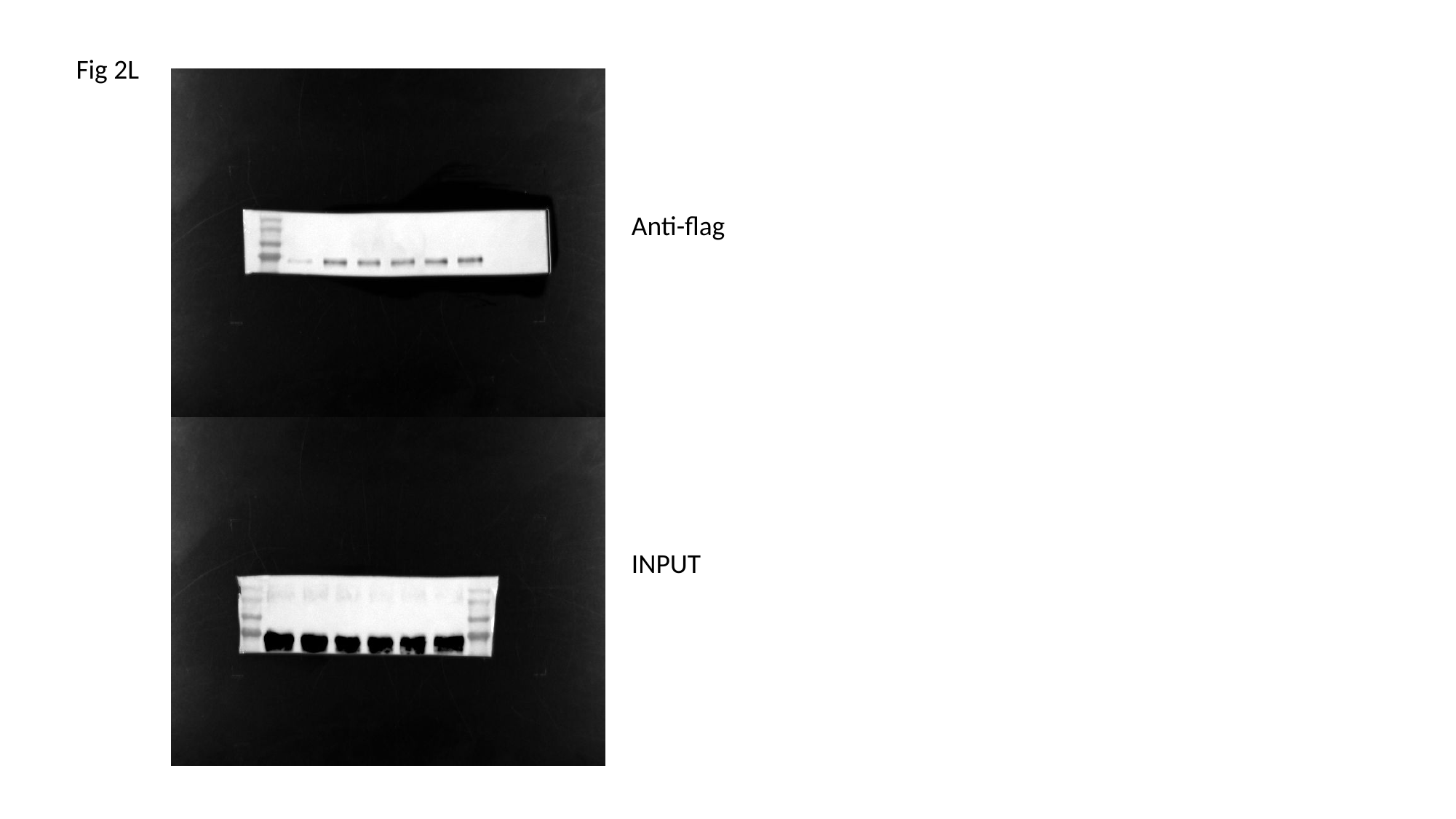

Fig 2L
Anti-flag
INPUT

## Slide 10
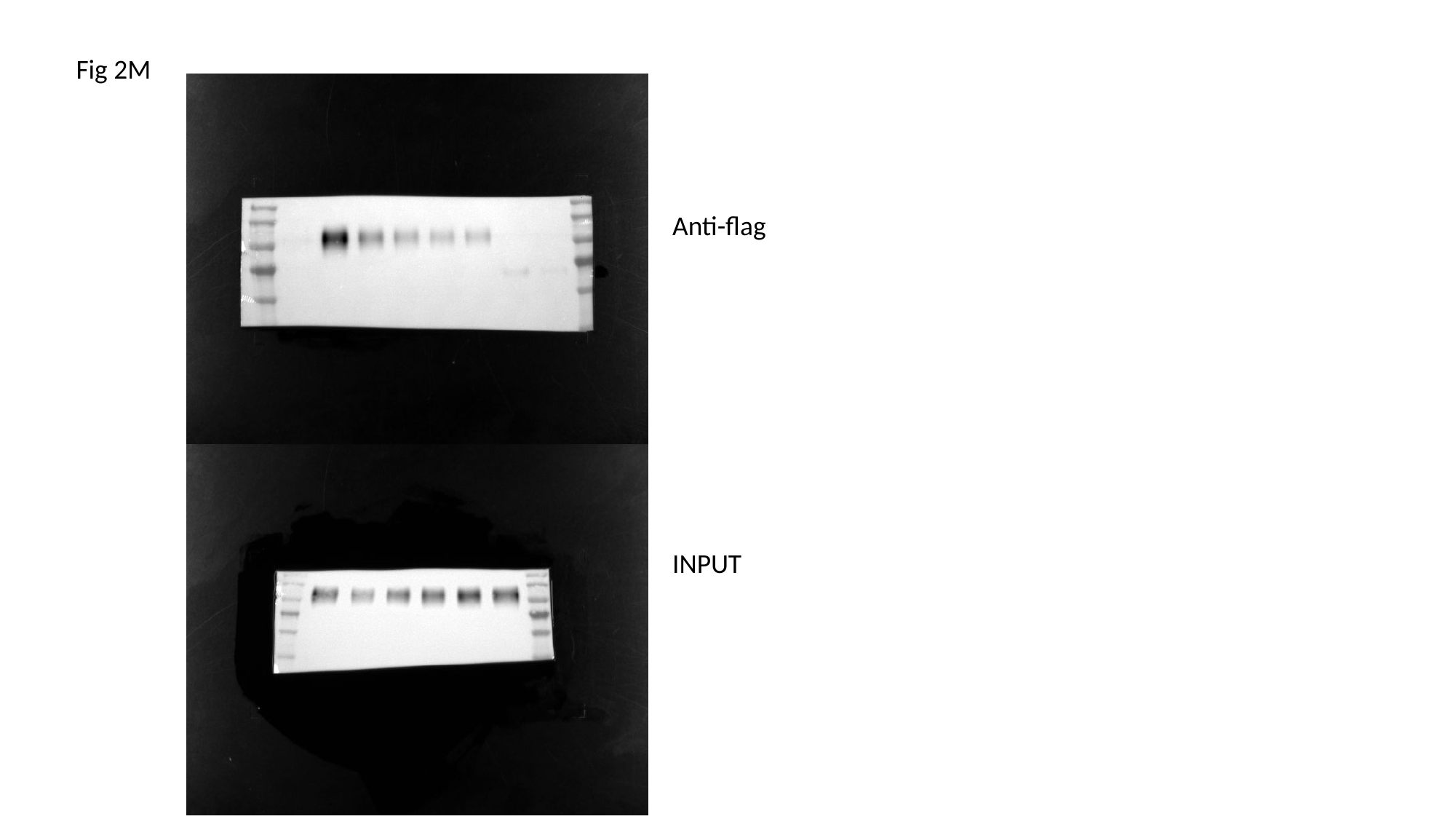

Fig 2M
Anti-flag
INPUT

## Slide 11
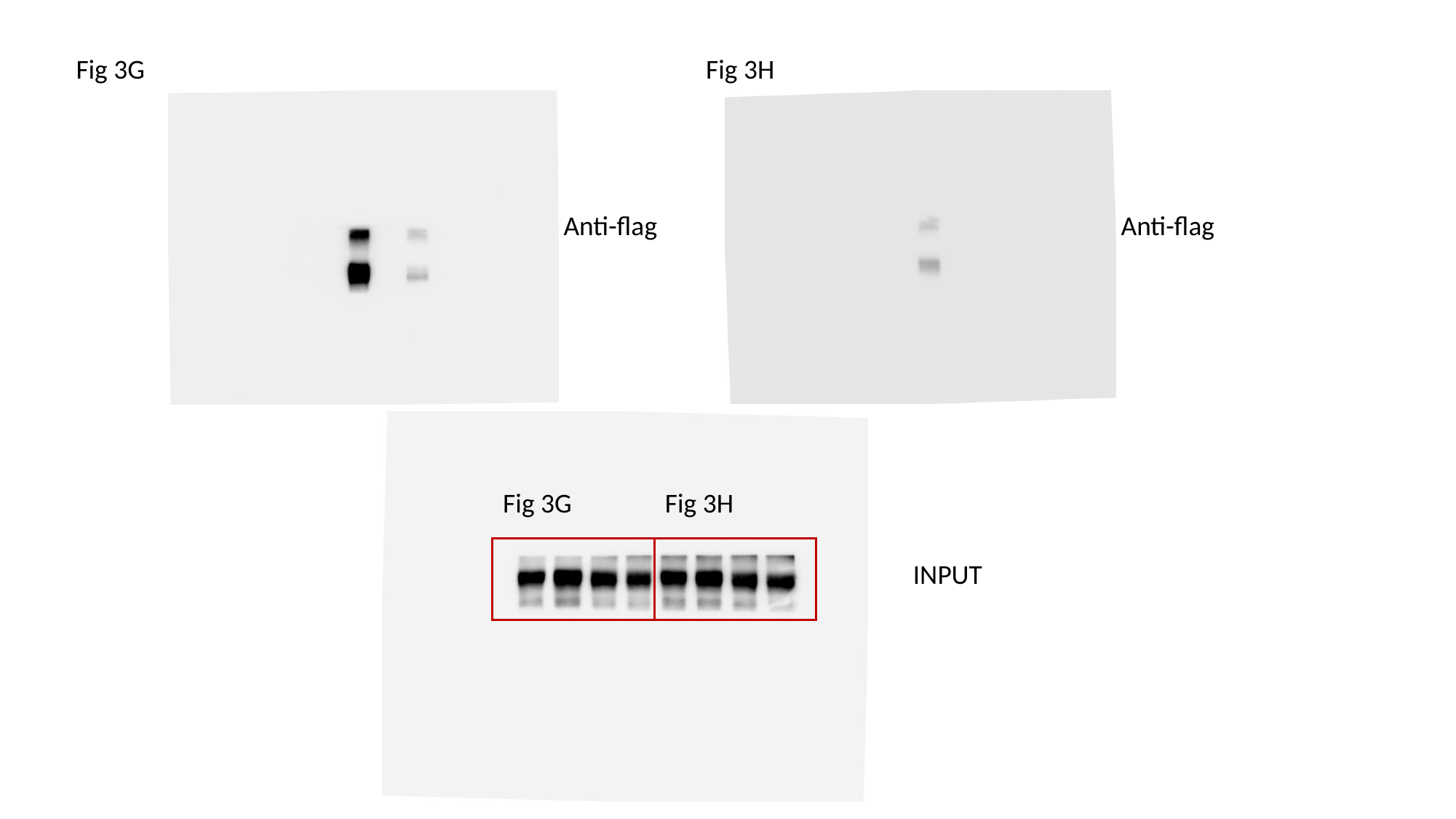

Fig 3G
Fig 3H
Anti-flag
Anti-flag
Fig 3G
Fig 3H
INPUT

## Slide 12
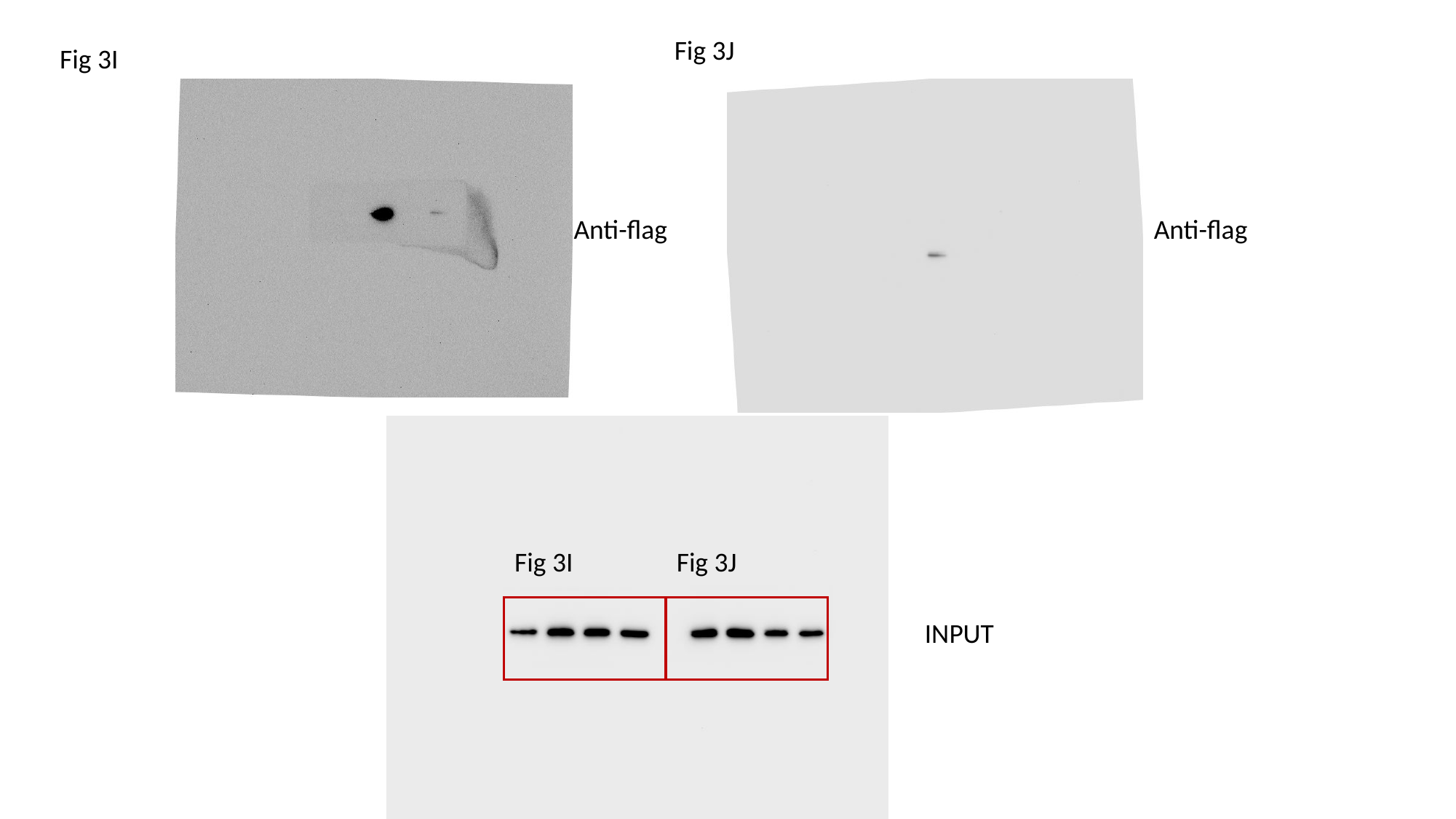

Fig 3J
Fig 3I
Anti-flag
Anti-flag
Fig 3I
Fig 3J
INPUT

## Slide 13
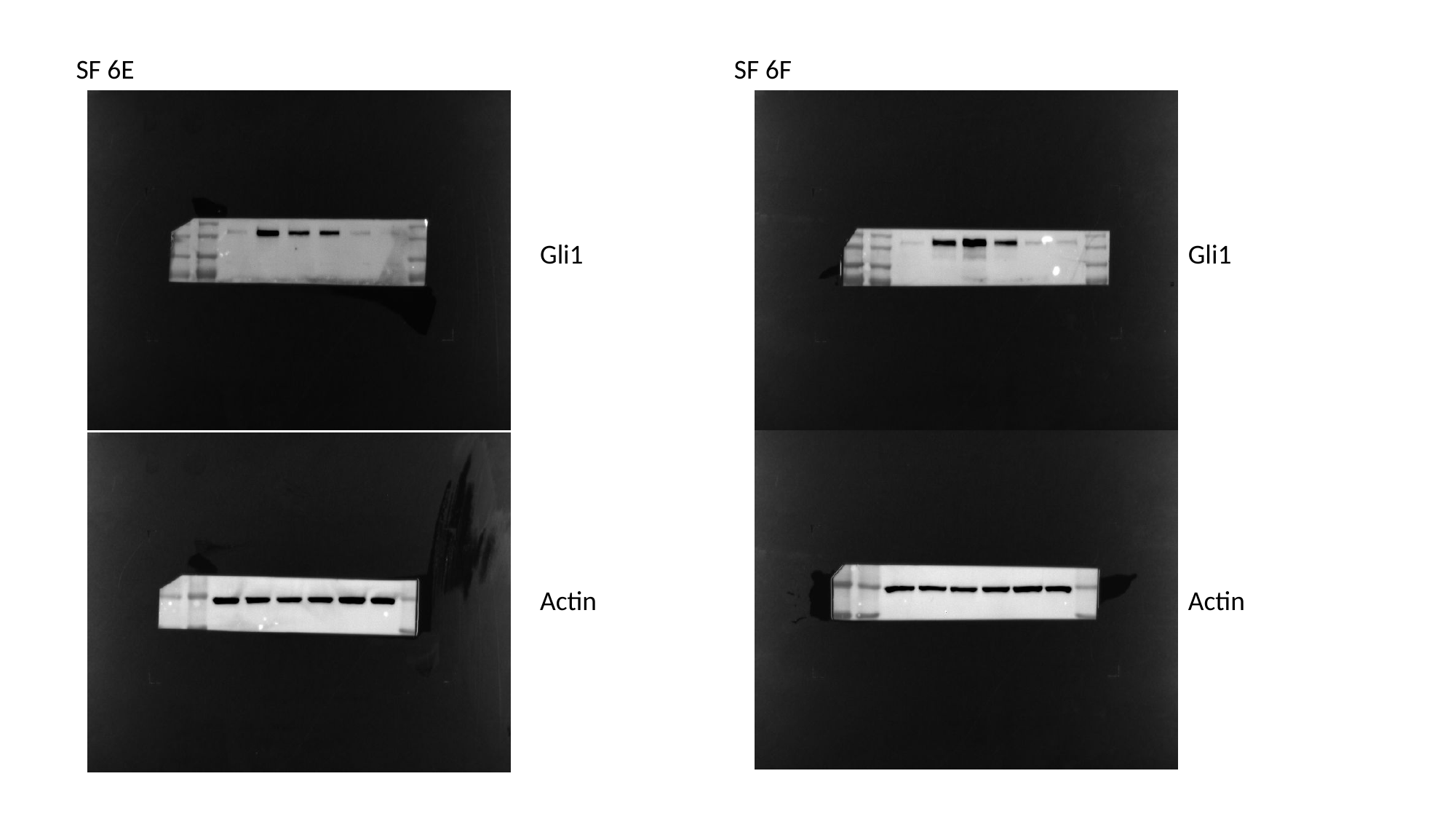

SF 6E
SF 6F
Gli1
Gli1
Actin
Actin
