# Supplementary material for: Cortisol regulates neonatal lung development via Smoothened
Source: Respir Res. 2025 Jan 18;26:27. doi: 10.1186/s12931-025-03104-0 (PMC11743026; doi:10.1186/s12931-025-03104-0)
Supplement: Supplementary file 3 — Supplementary Material 3 [file 12931_2025_3104_MOESM3_ESM.docx]

Supplemental Figures

**
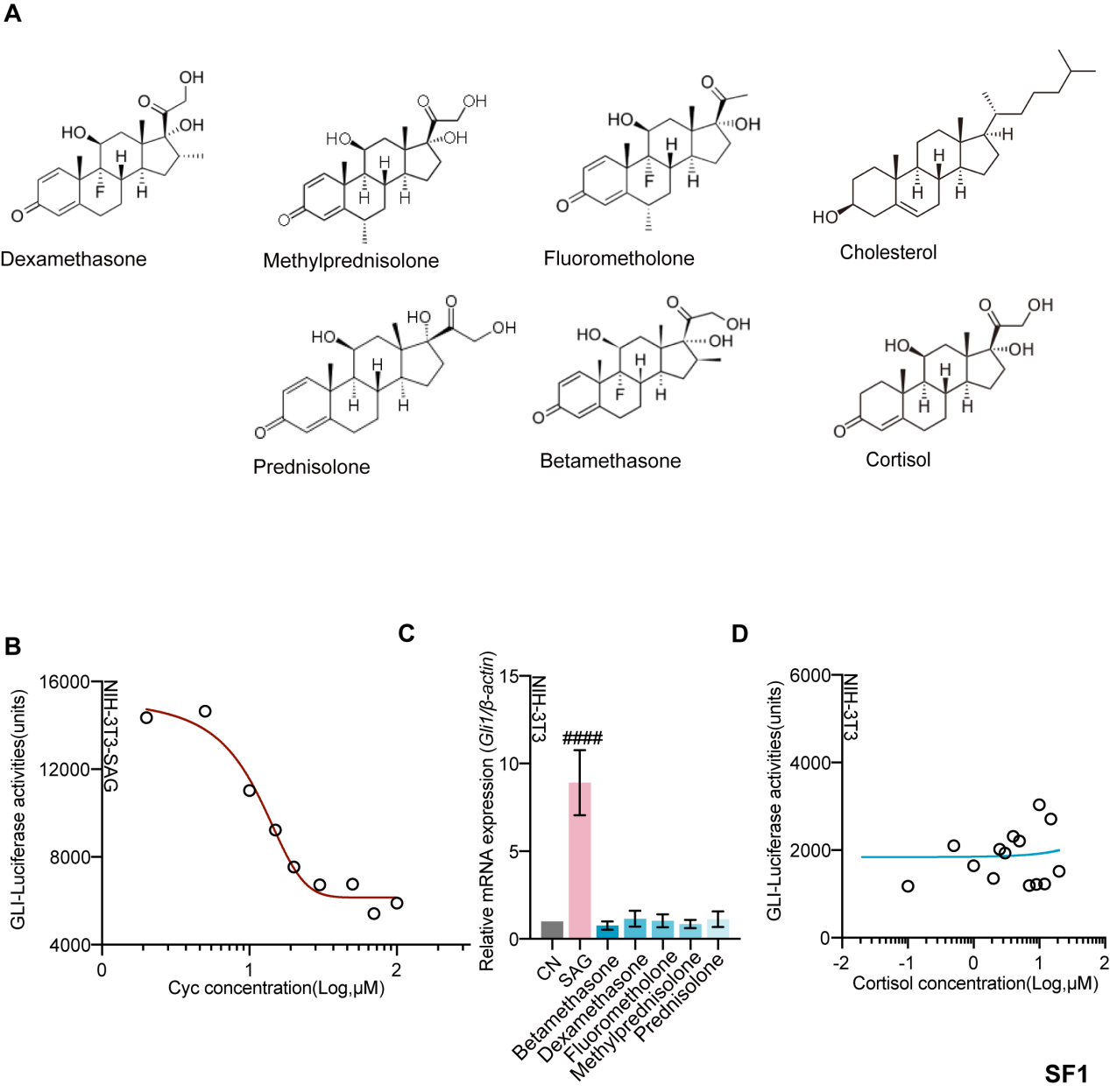
**

**Supplementary Fig. 1** | **Cortisol inhibited activation of SHH pathway, related to Fig. 1. A,** Structure of synthesized glucocorticoids (GCs) and cortisol. **B,** Luciferase activity in NIH-3T3 cells transfected with *Gli*-luciferase reporter treated with SAG in the presence of Cyc at indicated concentrations for 30 hours, n = 3. **C,** Relative mRNA expression of *Gli1* in NIH-3T3 cells in the presence of the indicated agents, n = 4. **D,** Luciferase activity in NIH-3T3 cells transfected with GLI*-*luciferase reporter in the presence of cortisol at indicated concentrations, n = 3.Unless stated, SAG, Smoothened agonist, 3 μM; Cyc, cyclopamine, 10 μM; Cort, cortisol, 1 μM; CN, control. #, compared with CN group; *, compared with SAG group. Data are represented as mean ± SEM. ** p < 0.01, **** p < 0.0001, #### p < 0.0001.


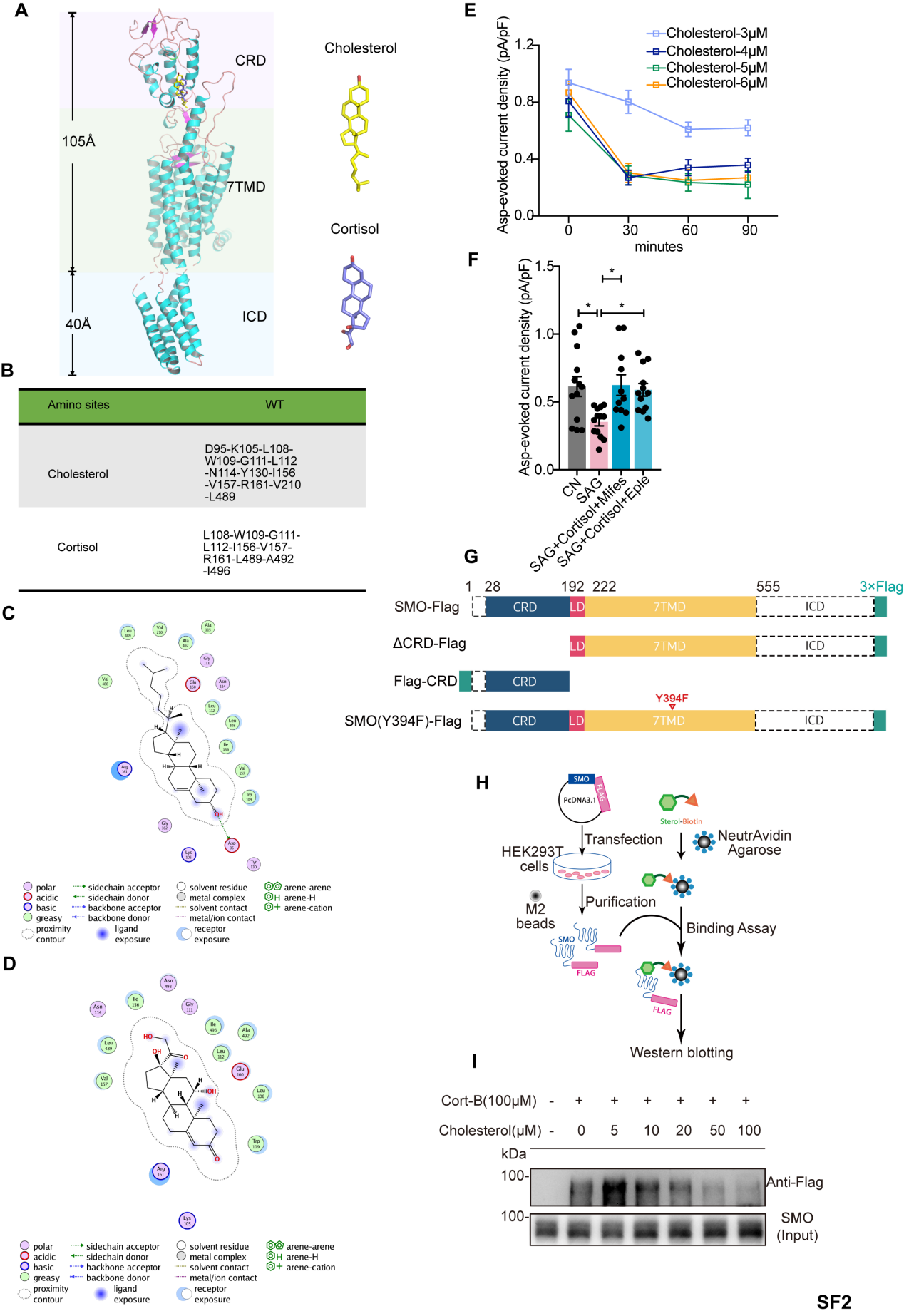


**Supplementary Fig. 2** | **Cortisol binding to SMO inhibited SHH pathway, related to Fig.2. A,** Overall structure showing the full length of human SMO, cholesterol, and cortisol in cartoon representation. Cortisol and cholesrerol binding to the cysteine-rich domain (CRD) domain of SMO predicted by molecular docking (PDB: 5l7d). **B-D**, Table of molecular simulation residues of cholesterol (**C**) and cortisol (**D**) in CRD binding groove. **E**, Asp-evoked current density in cultured neurons in the presence of cholesterol at the indicated times and concentrations, n = 10-16. **F**,Asp-evoked current density in cultured neurons incubated with SAG and cortisol in presence of mifepristone and eplerenone, n = 11-13. **G**, Schematics of different truncated fragments of SMO with flag tag. **H**, Schematics of in vitro binding experiments. **I,** Purified SMO binding to biotinylated cortisol and free cholesterol. SMO captured on cortisol beads in the presence of increasing concentrations of free cholesterol. Unless stated, CRD, cysteine-rich domain; 7TMD, seven-transmembrane domain, ICD, intracellular domain; WT, wild type; Cholesterol, 5 μM. Data are represented as mean ± SEM. ** p < 0.05; *** p < 0.001.


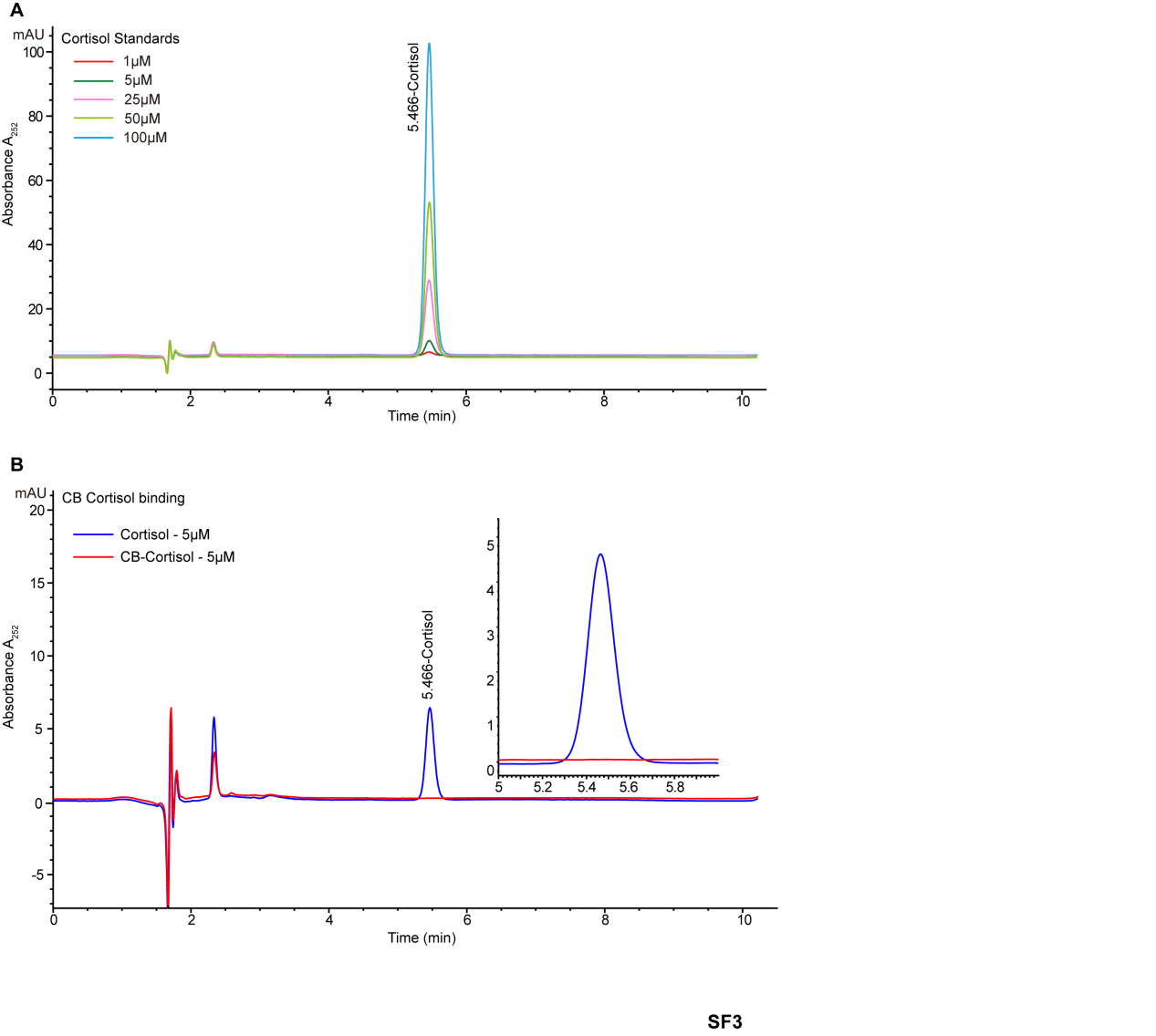


**Supplementary Fig. 3** | **HPLC analysis of cortisol, related to Fig.3. A and B,** Cortisol peaks were identified based on the retention time of the standards and confirmed by comparison of the 242 nm wavelength scan spectra. Cortisol standards (**A**) and cortisol incubated with biotinylated cholesterol beads *in vitro,* washed (**B)** and analyzed by HPLC in high-salt solution. Blue line, cortisol; red line, supernatant of the binding of cortisol to CB.


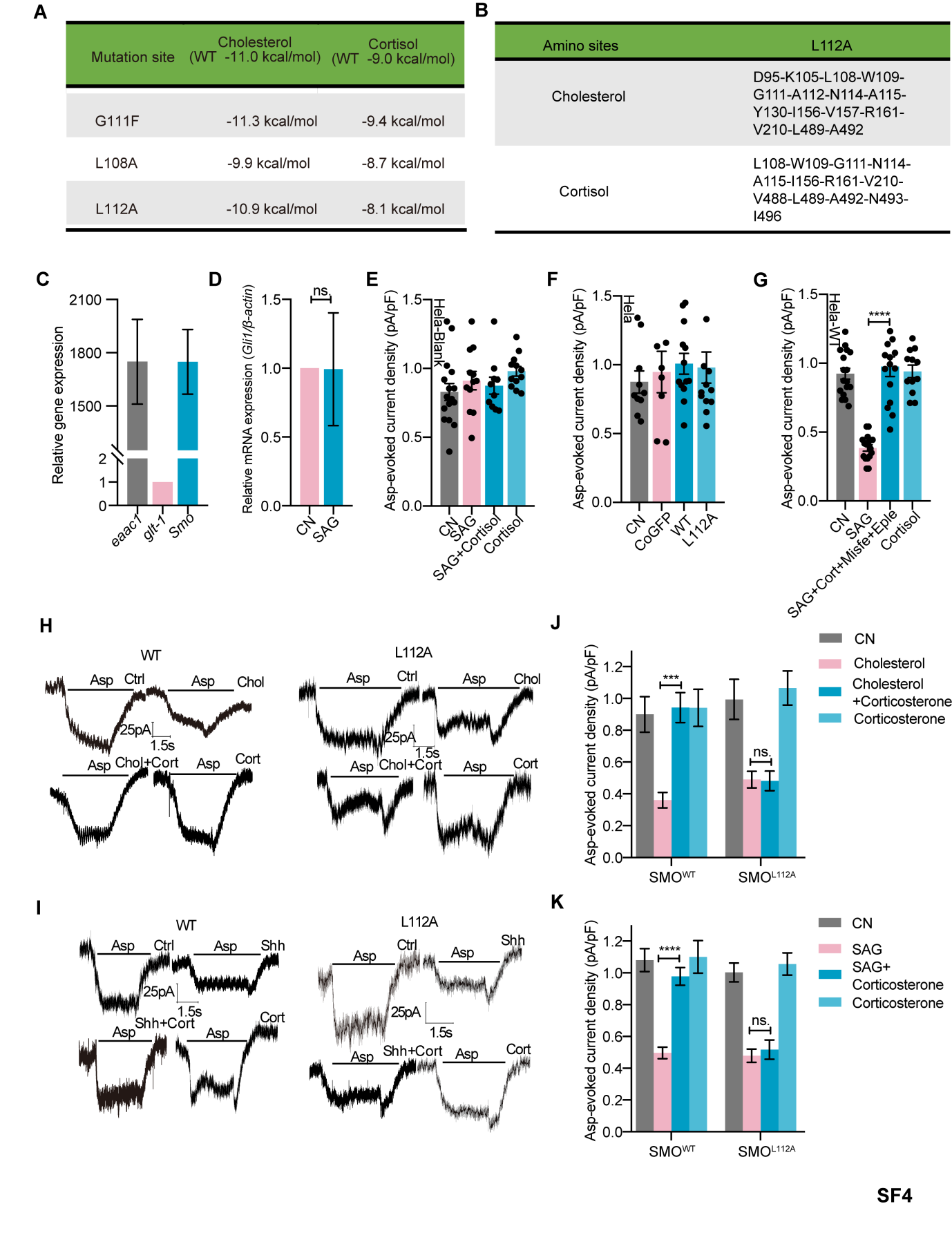


**Supplementary Fig. 4** | **L112 of SMO was crucial for cortisol inhibition for SHH pathway, related to Fig.3. A,** Table of molecular docking energy of cholesterol or cortisol binding to mutant SMO at different amino acid sites. **B,** Table of molecular simulation residues of cortisol and cholesterol in CRD binding groove. **C,** Gene expression of *eaac1, glt-1* and *Smo* in Hela cells, n = 7. **D,** Relative mRNA expression of *Gli1* in Hela cells in the presence of the indicated agents, n = 8. **E,** Asp-evoked current density in Hela cells in the presence of indicated reagents, n = 10-16. **F** and **G,** Asp-evoked current density in Hela cells transfected with SMO variants at indicated conditions (**F**, n = 8-13). Asp-evoked current density in Hela cells transfected with SMO (WT) in the presence indicated reagents (**G**, n = 12-17). **H** and **I,** Representative traces of Asp-evoked current in Hela cells transfected with SMO WT or L112A mutant in the presence of cholesterol (**H**) or Shh (**I**) and cortisol. **J** and **K,** Asp-evoked current density in Hela cells transfected with SMO WT and L112A incubated with cholesterol (**J**, n = 7-11) or SAG (**K**, n = 7-17) and in the presence of hydrocortisone. Unless stated, Chol, cholesterol; WT, SMO^WT^; L112A, SMO^L112A^; CoGFP: pcdh-CoGFP. Data are represented as mean ± SEM. *** p < 0.001; **** p < 0.0001; ns., no significance, p > 0.05.


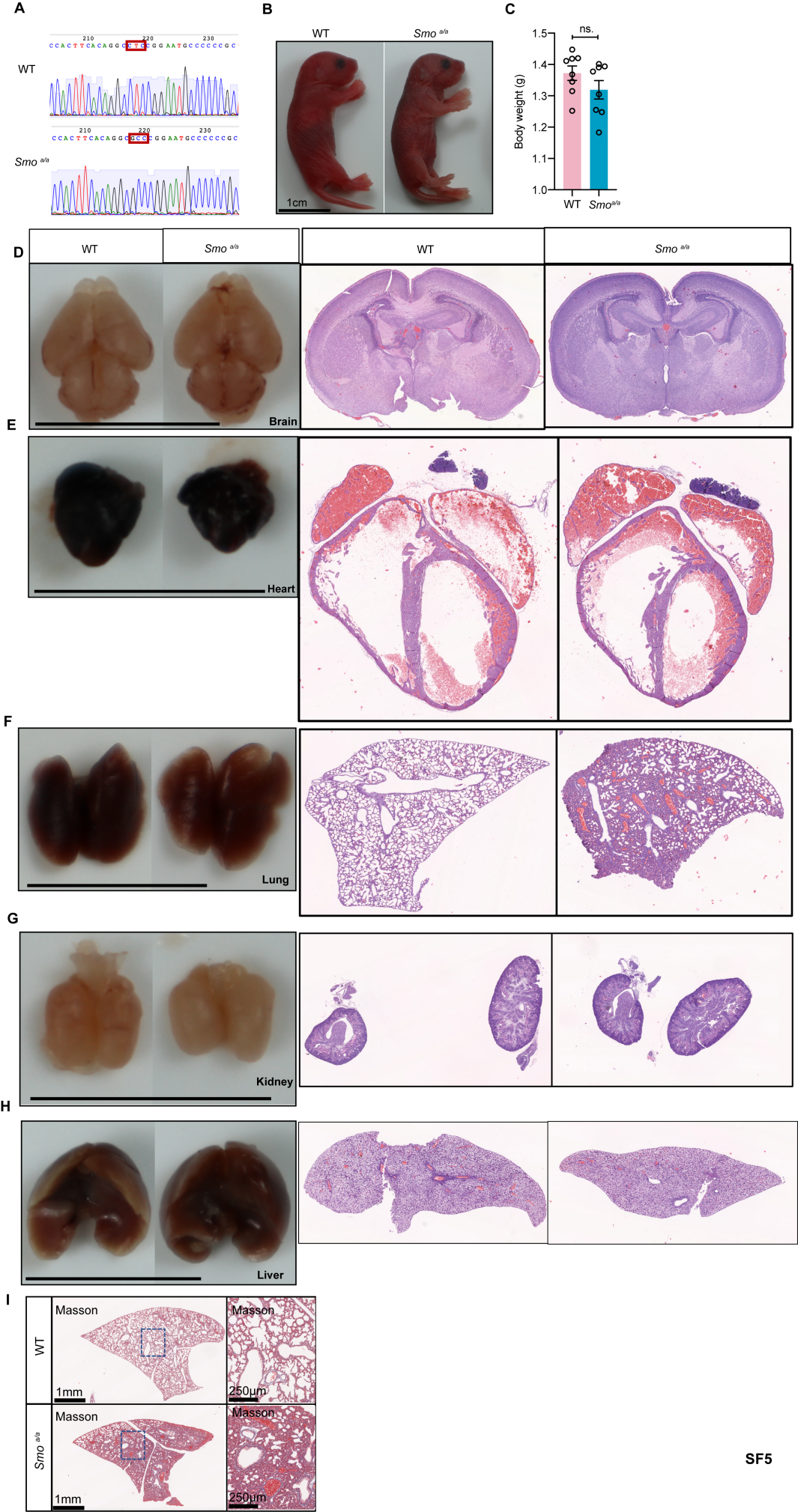


**Supplementary Fig. 5** | **Organizational morphology of WT and *Smo^a/a^* mice, related to Fig. 5. A,** Genotype sequencing of WT and *Smo^a/a^*(L116A) mice. **B,** Shape, size and skin color of neonatal WT and *Smo^a/a^* pups. Scale bar = 1cm. **C,** The statistics of weight of P0 WT and *Smo^a/a^* mice, n = 8. **D-H,** Anatomy of brain (**D**), heart (**E**), lung (**F**), kidney (**G**) and liver (**H**) from WT and *Smo^a/a^* mice at P0. Scale bar = 1cm. **I,** Typical images of lung sections with Masson-stained from newborn WT and *Smo^a/a^* mice. Left scale bar = 1mm, right scale bar = 250μm. Data are represented as mean ± SEM. ns., no significance, p > 0.05.


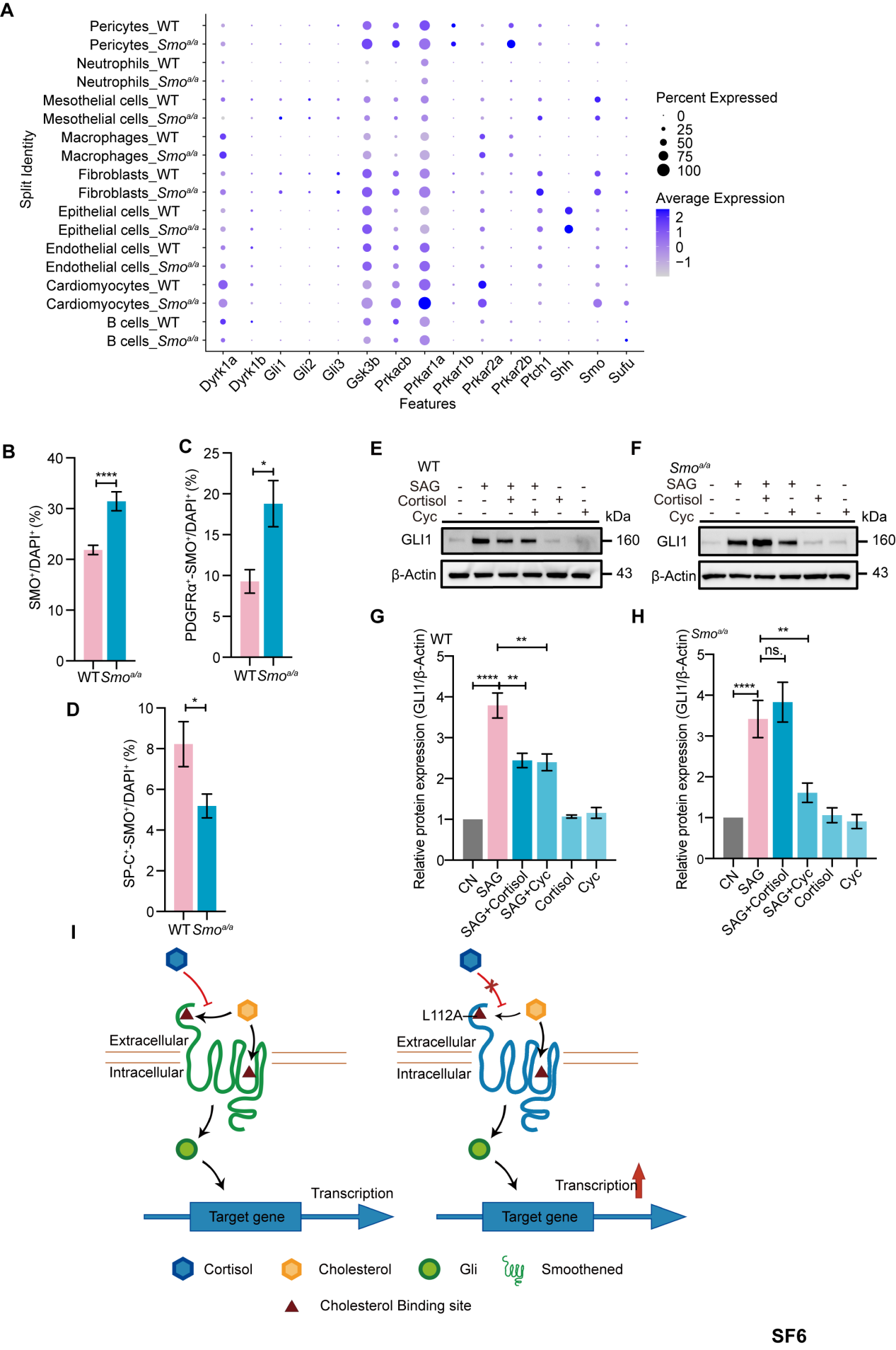


**Supplementary Fig. 6** | **Statistical analysis of WT and *Smo^a/a^* mice on newborn, related to Fig.5and Fig6.A,** plot of SHH pathway features in cell clusters. **B-D,** Statistics of FISH staining SMO-probe positive cells (**B**, n = 13 and 26 from 6 mice) and co-label with PDGFRα-positive (**C**, n = 6 and 11 from 3 mice) or prosurfactant protein C-positive cells (**D**, n = 7 and 14 from 4 mice) in lung sections. **E-H,** Representative immunoblots of total lysates extracted from the WT (**E**) or *Smo^a/a^* (**F**) MEFs in the presence of the indicated agents using the indicated antibodies. Statistics of immunoblots of total lysates extracted for Supplementary Fig.6e (**G**, n = 3) and Supplementary Fig.6f (**H**, n = 4). **I**, Diagram of cortisol acts on Smoothened. All western blots performed three or more replicates. Data are represented as mean ± SEM. * p < 0.01; ** p < 0.05; *** p < 0.001; **** p < 0.0001; ns., no significance, p > 0.05.
