# Supplementary figures and images for: Cortisol regulates neonatal lung development via Smoothened
Source: Respir Res. 2025 Jan 18;26:27. doi: 10.1186/s12931-025-03104-0 (PMC11743026; doi:10.1186/s12931-025-03104-0)

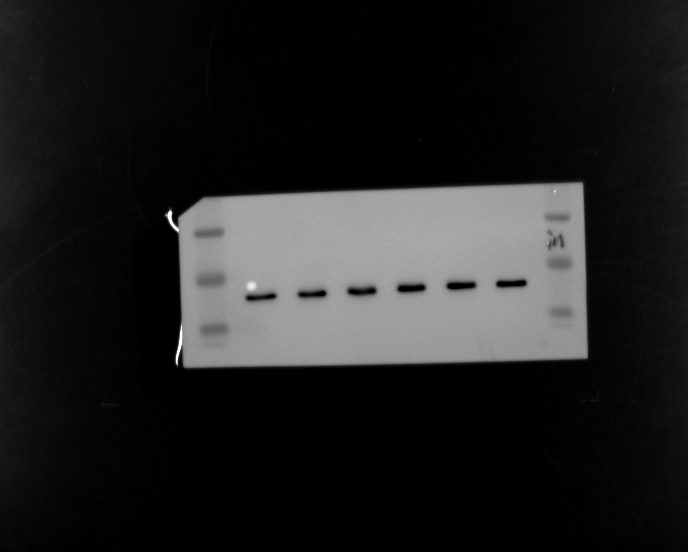

Supplement: Supplementary file 4 — Supplementary Material 4 [file 12931_2025_3104_MOESM4_ESM.zip › origonal data/Fig 2H-INPUT-CHOLESTEROL-CRD-0-5-10-20-50-100-M.tif]

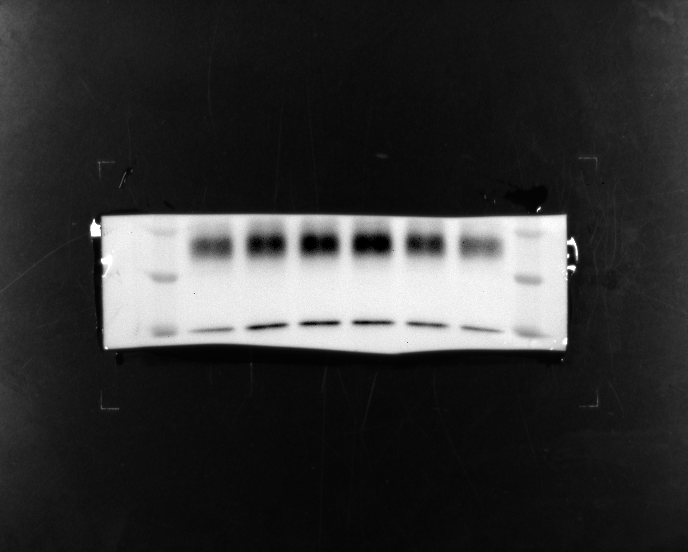

Supplement: Supplementary file 4 — Supplementary Material 4 [file 12931_2025_3104_MOESM4_ESM.zip › origonal data/Fig 2J-INPUT-CRD-MW.tif]

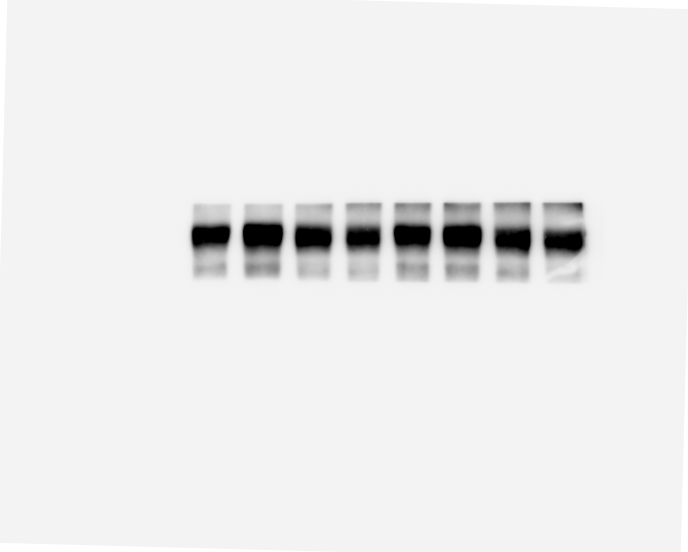

Supplement: Supplementary file 4 — Supplementary Material 4 [file 12931_2025_3104_MOESM4_ESM.zip › origonal data/Fig 3G and 3H-INPUT-WT-L112A.tif]

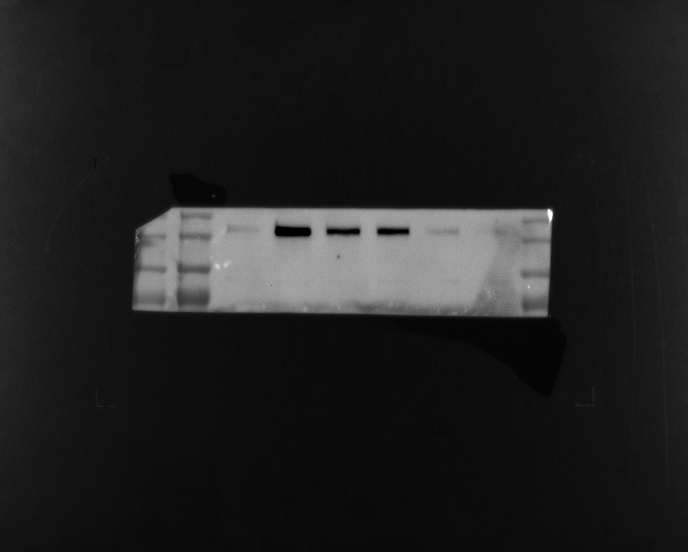

Supplement: Supplementary file 4 — Supplementary Material 4 [file 12931_2025_3104_MOESM4_ESM.zip › origonal data/SF 6E GLI1-MEF-GLI-1-WT-CN-SAG-S+CORT-S+CYC-CORT-CYC.tif]

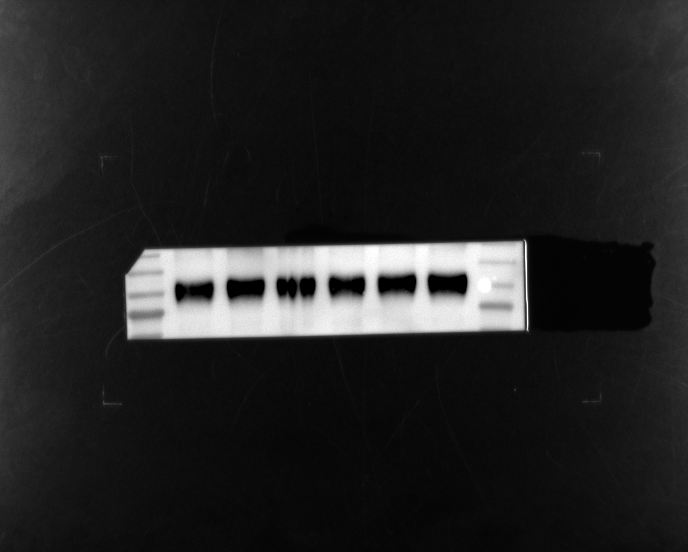

Supplement: Supplementary file 4 — Supplementary Material 4 [file 12931_2025_3104_MOESM4_ESM.zip › origonal data/Fig 2F-INPUT-0-1-5-10-20-40-I-M-M.tif]

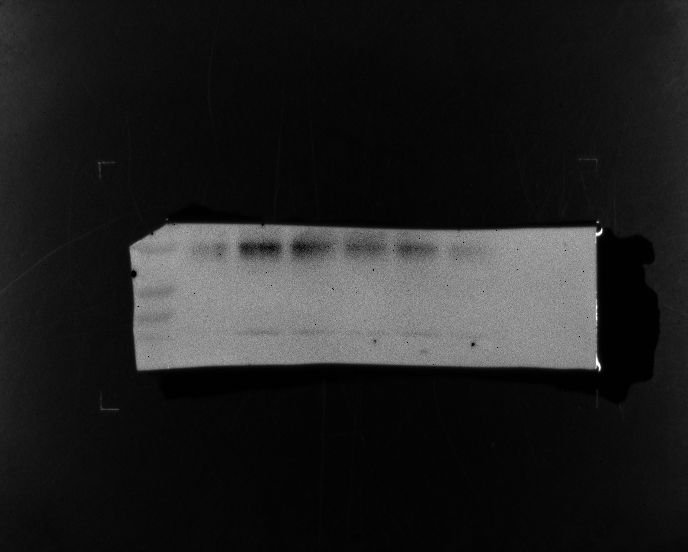

Supplement: Supplementary file 4 — Supplementary Material 4 [file 12931_2025_3104_MOESM4_ESM.zip › origonal data/Fig 2J-anti flag-CRD-MW.tif]

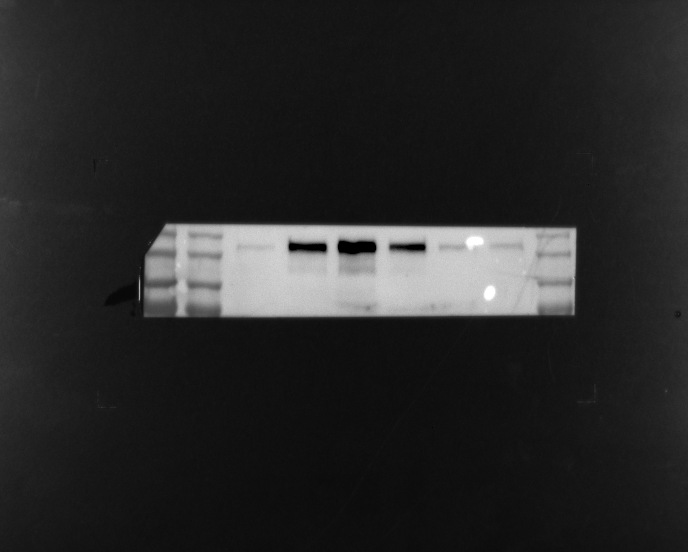

Supplement: Supplementary file 4 — Supplementary Material 4 [file 12931_2025_3104_MOESM4_ESM.zip › origonal data/SF 6F-Gli1-MEF-116-CN-SAG-S+CORT-S+CYC-CORT-CYC.tif]

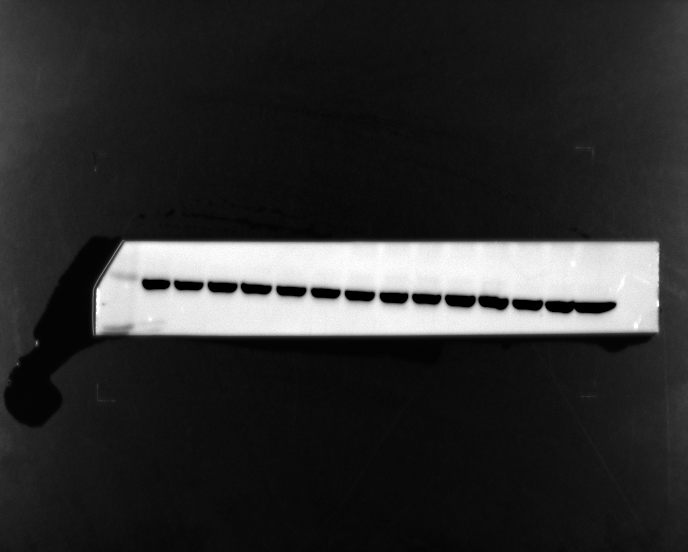

Supplement: Supplementary file 4 — Supplementary Material 4 [file 12931_2025_3104_MOESM4_ESM.zip › origonal data/Fig 1C-actin.tif]

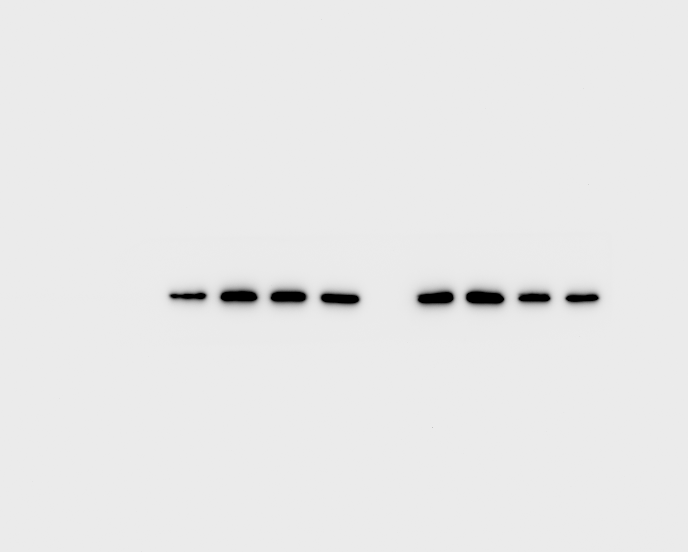

Supplement: Supplementary file 4 — Supplementary Material 4 [file 12931_2025_3104_MOESM4_ESM.zip › origonal data/Fig 3I and 3J-INPUT.tif]

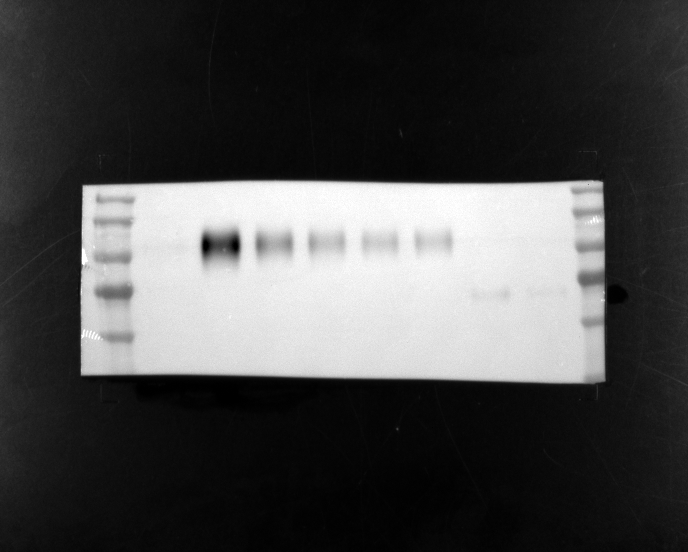

Supplement: Supplementary file 4 — Supplementary Material 4 [file 12931_2025_3104_MOESM4_ESM.zip › origonal data/Fig 2M-anti flag-Y394F--MW.tif]

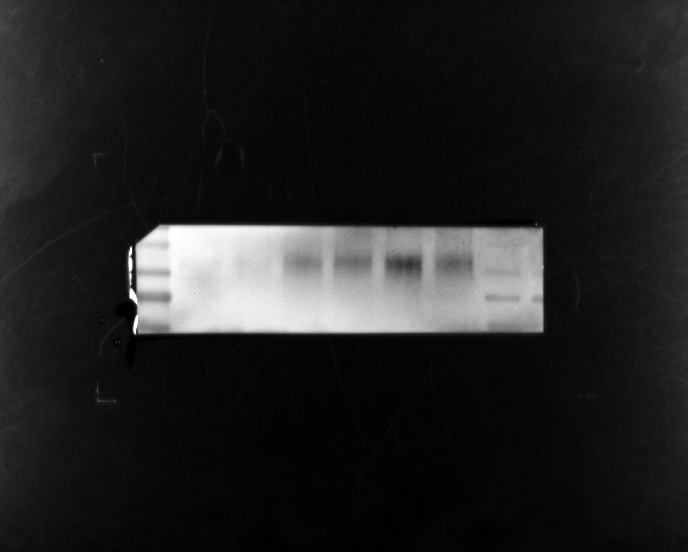

Supplement: Supplementary file 4 — Supplementary Material 4 [file 12931_2025_3104_MOESM4_ESM.zip › origonal data/Fig 2F-anti flag-0-1-5-10-20-40-CORTISOL-5MIN-S-M.tif]

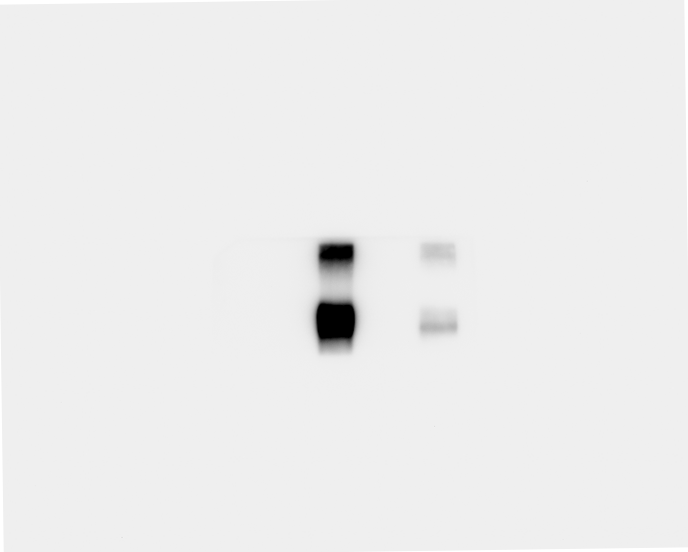

Supplement: Supplementary file 4 — Supplementary Material 4 [file 12931_2025_3104_MOESM4_ESM.zip › origonal data/Fig 3G-anti flag-WT-L112A-CN-CB-LL.tif]

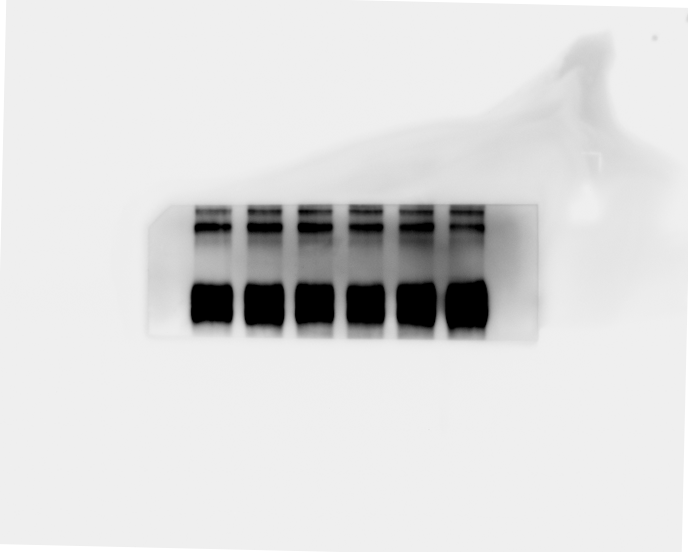

Supplement: Supplementary file 4 — Supplementary Material 4 [file 12931_2025_3104_MOESM4_ESM.zip › origonal data/Fig 2E-INPUT-CHOLESTEROL-BINDING-0-1-5-10-20-40-L-INPUT.tif]

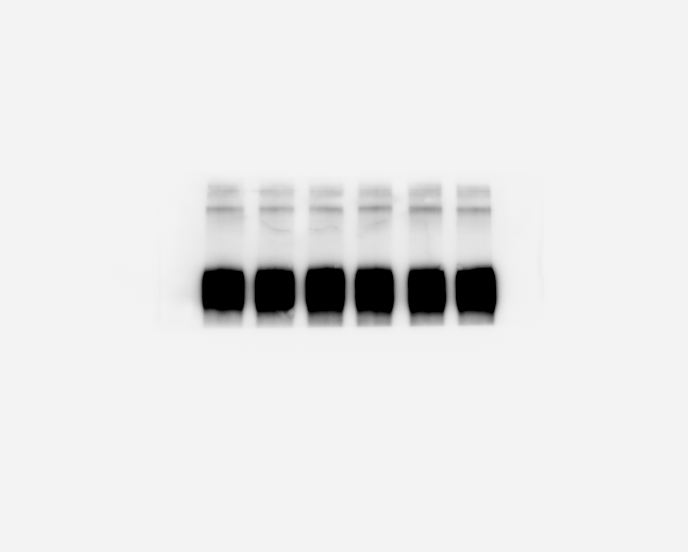

Supplement: Supplementary file 4 — Supplementary Material 4 [file 12931_2025_3104_MOESM4_ESM.zip › origonal data/Fig 2G-INPUT-CORTISOL-0-10-20-100-200-INPUT-L.tif]

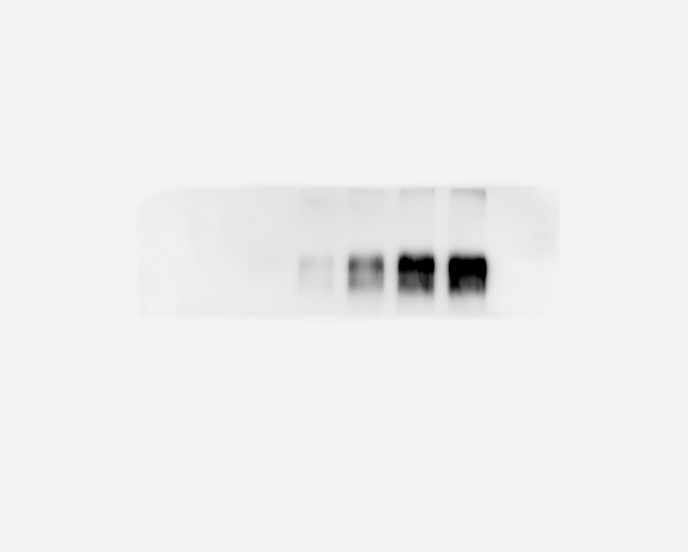

Supplement: Supplementary file 4 — Supplementary Material 4 [file 12931_2025_3104_MOESM4_ESM.zip › origonal data/Fig 2E-anti flag-CHOLESTEROL-BINDING-0-1-5-10-20-40-M.tif]

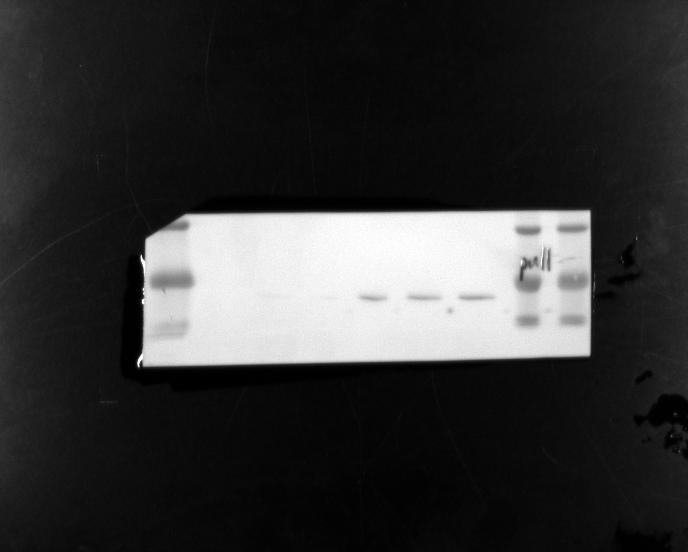

Supplement: Supplementary file 4 — Supplementary Material 4 [file 12931_2025_3104_MOESM4_ESM.zip › origonal data/Fig 2I-anti flag-PULLDOWN-0-5-10-20-50-100-M.tif]

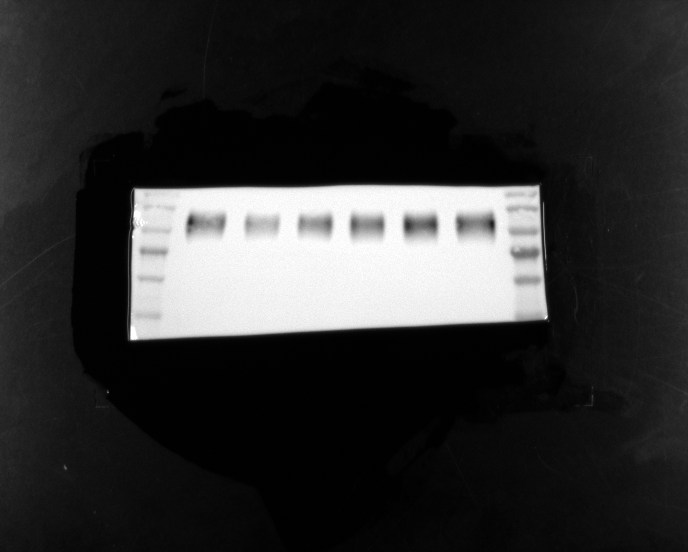

Supplement: Supplementary file 4 — Supplementary Material 4 [file 12931_2025_3104_MOESM4_ESM.zip › origonal data/Fig 2M-INPUT-Y394F-MW.tif]

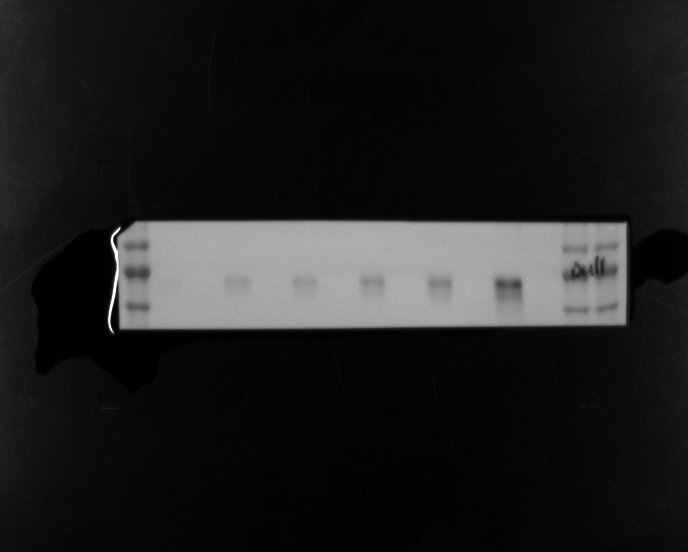

Supplement: Supplementary file 4 — Supplementary Material 4 [file 12931_2025_3104_MOESM4_ESM.zip › origonal data/Fig 2K-anti flag.tif]

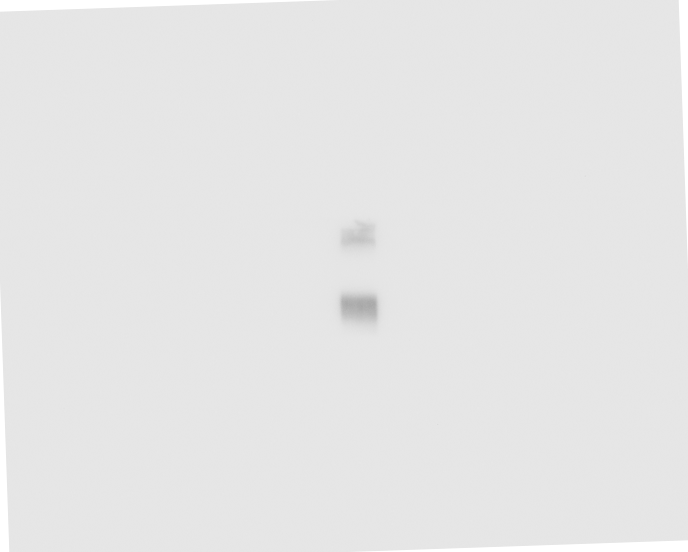

Supplement: Supplementary file 4 — Supplementary Material 4 [file 12931_2025_3104_MOESM4_ESM.zip › origonal data/Fig 3H-anti flag-WT-L112A-CN-CORTB.tif]

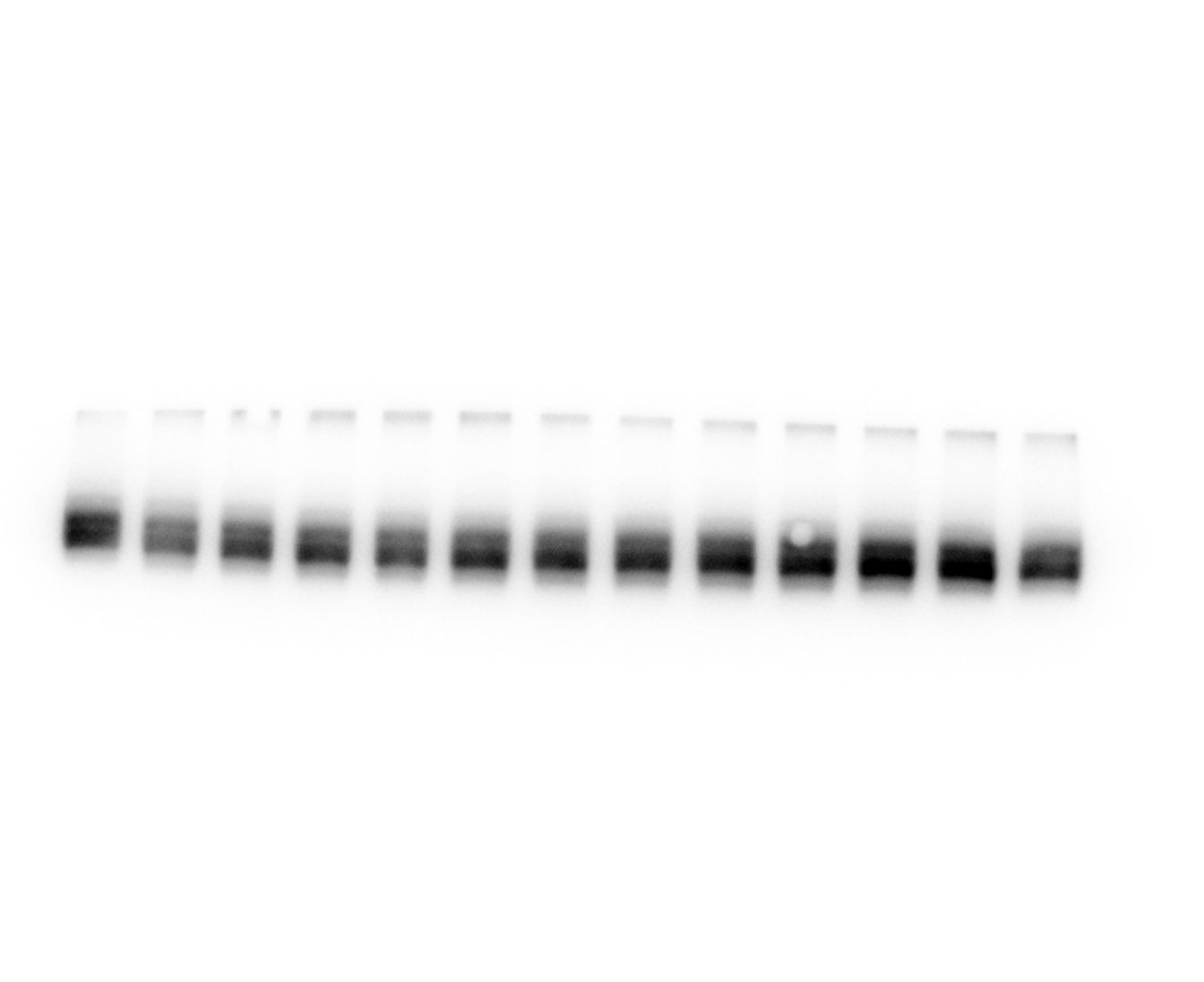

Supplement: Supplementary file 4 — Supplementary Material 4 [file 12931_2025_3104_MOESM4_ESM.zip › origonal data/SF 2I-INPUT.Tif]

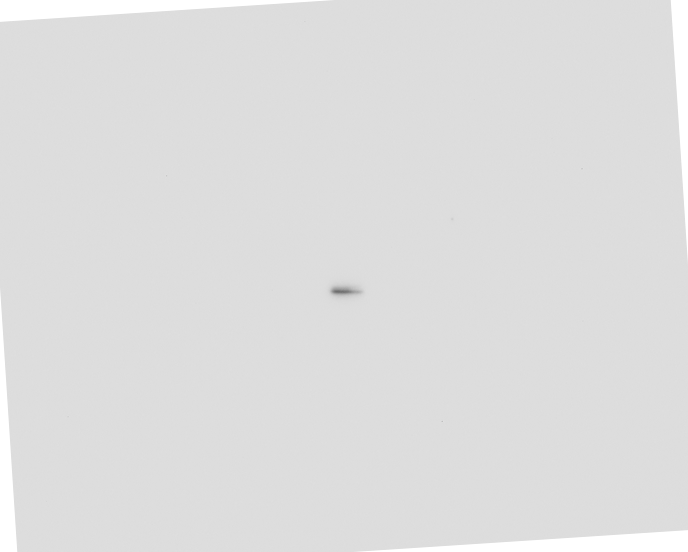

Supplement: Supplementary file 4 — Supplementary Material 4 [file 12931_2025_3104_MOESM4_ESM.zip › origonal data/Fig 3J-anti flag-CRD-CRDL112A-CN-CORTB-L.tif]

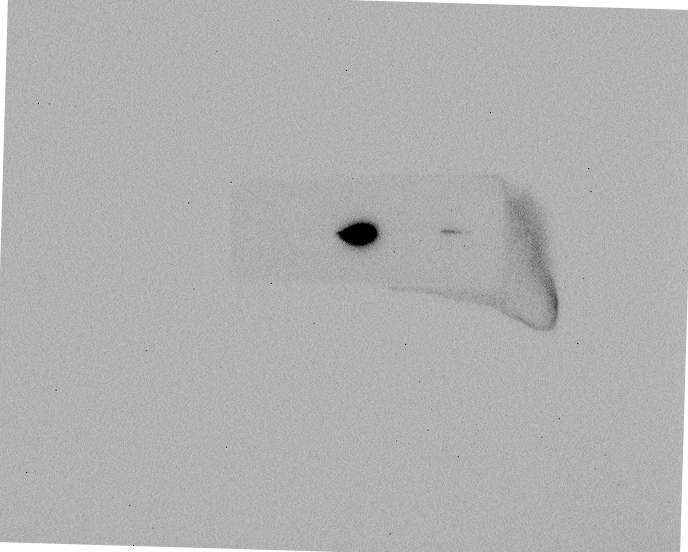

Supplement: Supplementary file 4 — Supplementary Material 4 [file 12931_2025_3104_MOESM4_ESM.zip › origonal data/Fig 3I-anti flag-CRD-CRDL112A-CN-CB-LL.tif]

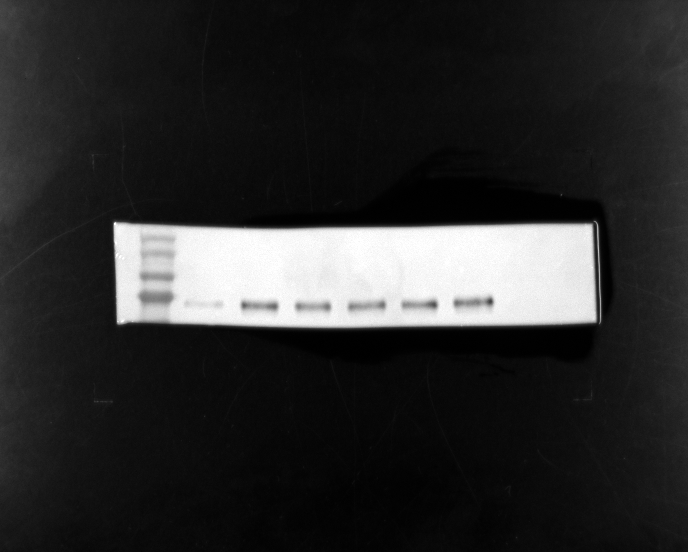

Supplement: Supplementary file 4 — Supplementary Material 4 [file 12931_2025_3104_MOESM4_ESM.zip › origonal data/Fig 2L-anti flag-dCRD-MW.tif]

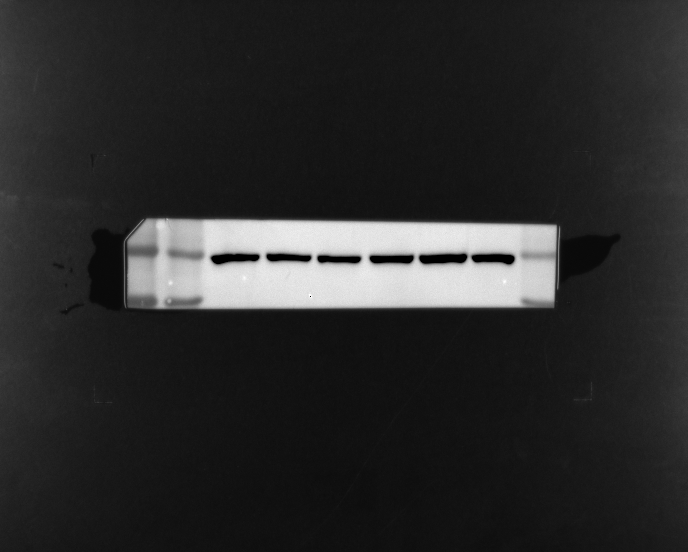

Supplement: Supplementary file 4 — Supplementary Material 4 [file 12931_2025_3104_MOESM4_ESM.zip › origonal data/SF 6F-actin-MEF-B-ACTIN-116-CN-SAG-S+CORT-S+CYC-CORT-CYC.tif]

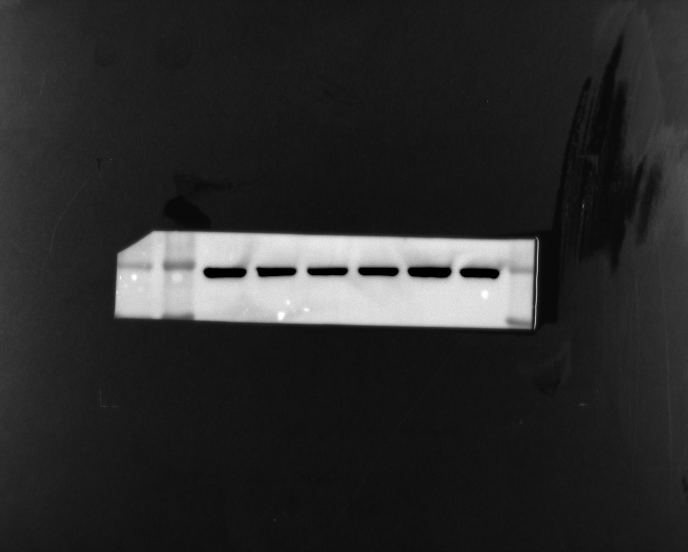

Supplement: Supplementary file 4 — Supplementary Material 4 [file 12931_2025_3104_MOESM4_ESM.zip › origonal data/SF 6E-actin-MEF-B-ACTIN-WT-CN-SAG-S+CORT-S+CYC-CORT-CYC-.tif]

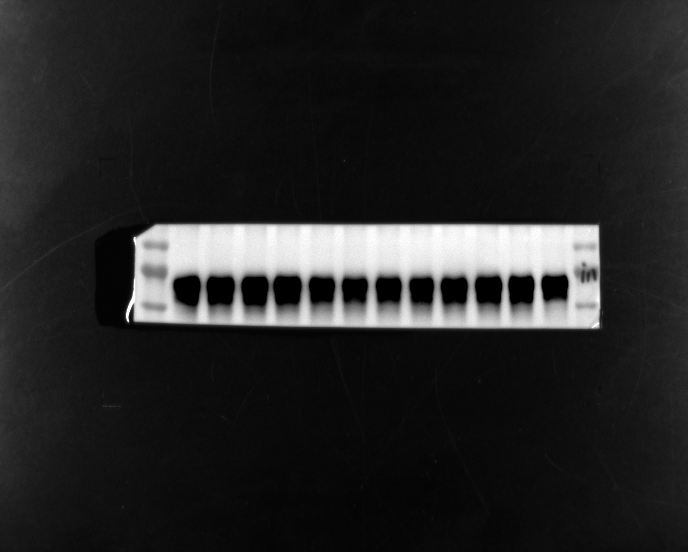

Supplement: Supplementary file 4 — Supplementary Material 4 [file 12931_2025_3104_MOESM4_ESM.zip › origonal data/Fig 2K-INPUT.tif]

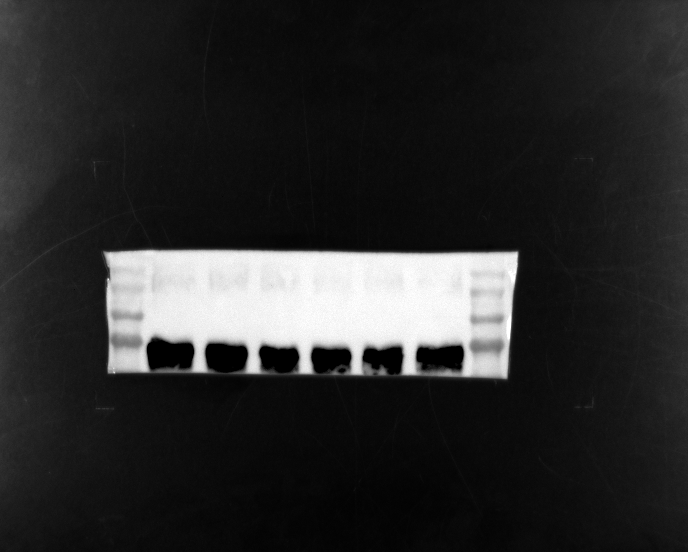

Supplement: Supplementary file 4 — Supplementary Material 4 [file 12931_2025_3104_MOESM4_ESM.zip › origonal data/Fig 2L-INPUT-dCRD-MW.tif]

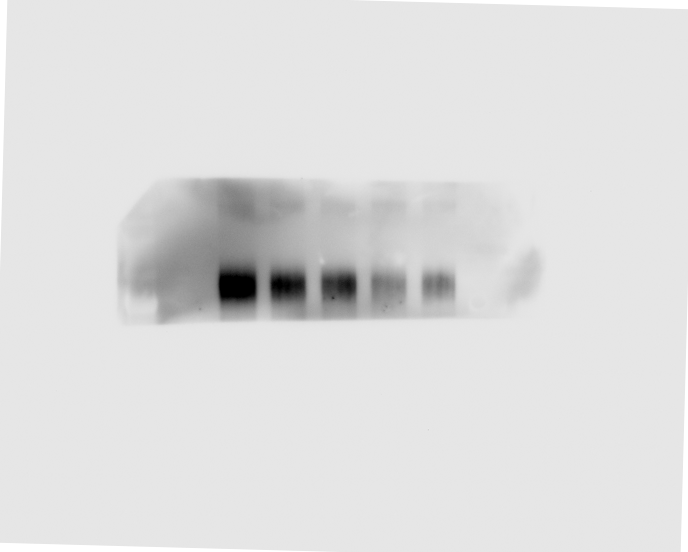

Supplement: Supplementary file 4 — Supplementary Material 4 [file 12931_2025_3104_MOESM4_ESM.zip › origonal data/Fig 2G-anti flag-CORTISOL-0-10-20-100-200-M.tif]

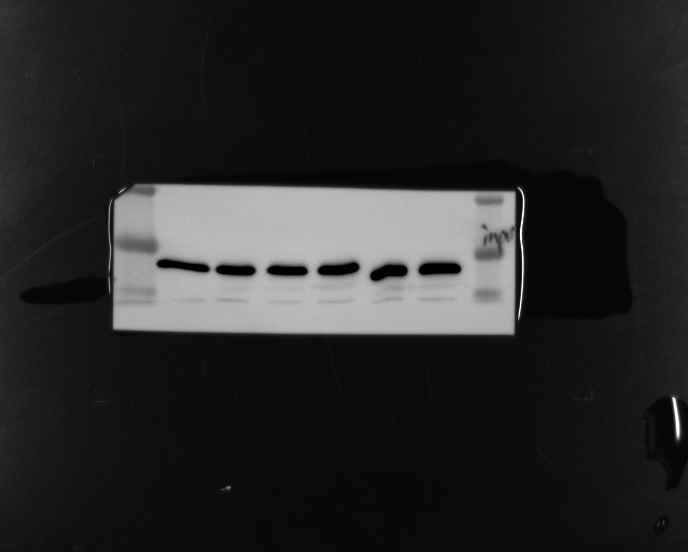

Supplement: Supplementary file 4 — Supplementary Material 4 [file 12931_2025_3104_MOESM4_ESM.zip › origonal data/Fig 2I-INPUT-0-5-10-20-50-100-M.tif]

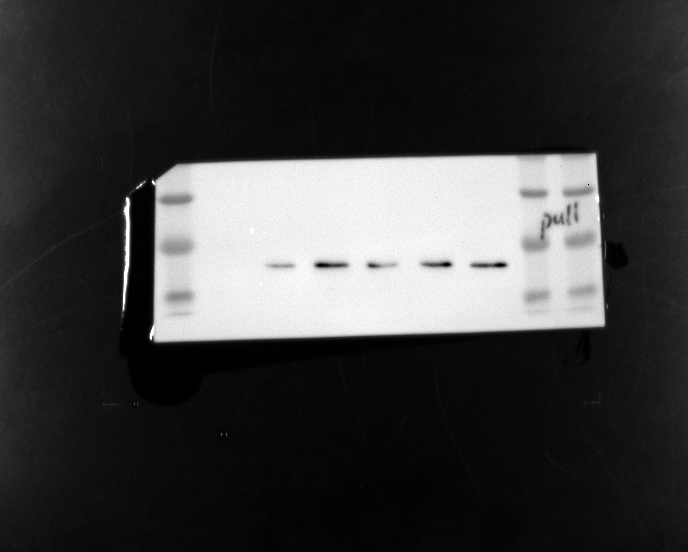

Supplement: Supplementary file 4 — Supplementary Material 4 [file 12931_2025_3104_MOESM4_ESM.zip › origonal data/Fig 2H-anti flag-PULL-DOWN-CHOLESTEROL-CRD-0-5-10-20-50-100-L-M.tif]

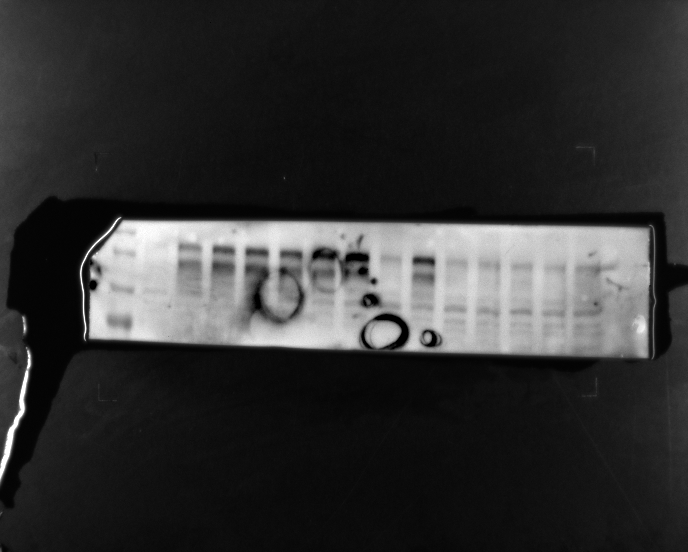

Supplement: Supplementary file 4 — Supplementary Material 4 [file 12931_2025_3104_MOESM4_ESM.zip › origonal data/Fig 1C-gli1.tif]

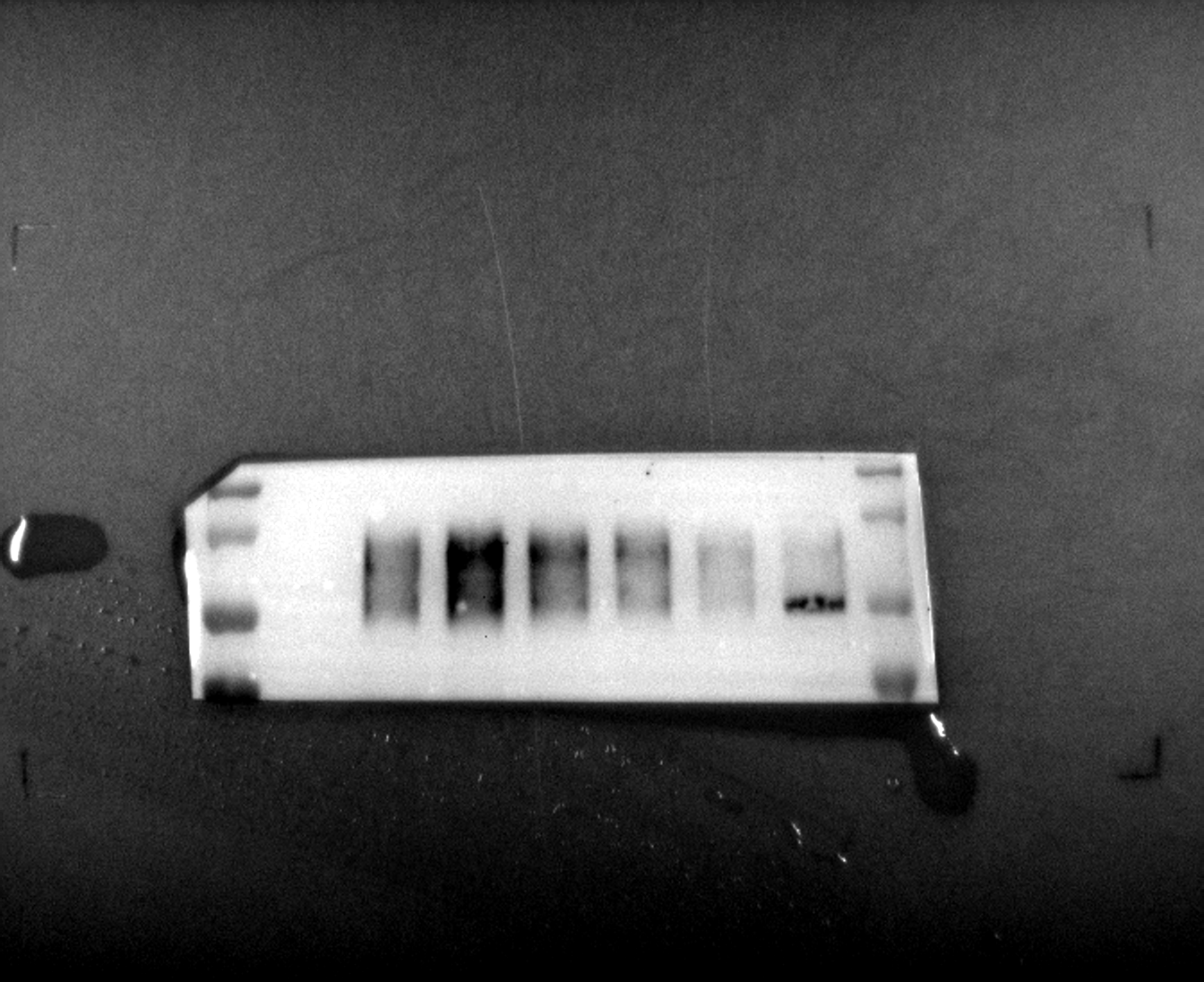

Supplement: Supplementary file 4 — Supplementary Material 4 [file 12931_2025_3104_MOESM4_ESM.zip › origonal data/SF 2I-anti flag.Tif]
